# The distribution and origin of Mercury’s crustal magnetization

**DOI:** 10.1073/pnas.2535723123

**Published:** 2026-08-17

**Authors:** Catherine L. Johnson, Alain M. Plattner, Filippo Cicchetti, Katarina Miljković

**Affiliations:** ^a^ Department of Earth, Ocean and Atmospheric Sciences, University of British Columbia, Vancouver, BC V6T 1Z4, Canada; ^b^ Planetary Science Institute, Tucson, AZ 85719; ^c^ Department of Geological Sciences, University of Alabama, Tuscaloosa, AL 35487; ^d^ School of Earth and Planetary Sciences, Space Science and Technology Centre, Curtin University, Perth, Western Australia 6102

**Keywords:** Mercury, crustal magnetism, dynamo history, impacts, volcanic plains

## Abstract

Mercury is the only planet other than Earth to possess both a global magnetic field that arises from a core dynamo, and a crustal magnetic field that arises from magnetized rocks. Crustal fields provide unique records of a planet’s interior state and evolution because magnetizations depend on the magnetizing field and the magnetic minerals present. A major unresolved issue for Mercury is the relative contributions of present-day (induced) and ancient (remanent) magnetizations—both are expected but are difficult to separate. The former can elucidate variations in crustal iron content and/or magnetic mineral properties, the latter can constrain the dynamo history. Here, we use crustal field observations from the MErcury, Surface, Space ENvironment, GEochemistry and Ranging mission to develop a magnetization model for Mercury’s northern latitudes. We use constraints on crustal thickness, magnetic mineralogy, iron content, and the present-day core field to assess the expected magnitudes of induced magnetizations. We find that these can fully explain the observed crustal fields at most places, and at some more strongly magnetized craters through impact-delivered iron. The strongest crustal fields, especially at Caloris, require a contribution from remanent magnetization and/or unusual magnetic mineral properties. Our results provide constraints on lateral variations in crustal iron content of both exogenic and endogenic origin. They also highlight a knowledge gap regarding the magnetic properties of Mercury’s crust, specifically its magnetic susceptibility, that has critical implications for understanding whether even the strongest magnetizations can elucidate Mercury’s dynamo history.

## Significance

Only Mercury and Earth have magnetic fields from a core dynamo and from crustal rocks that are magnetized in the present-day and/or past core field. Magnetized crustal rocks provide a window into a planet’s magnetic history and into the magnetic minerals and properties of its outermost layers. At Mercury, rocks are easily magnetized by its present-day core field, even though that field is 100× weaker than Earth’s. In most places, the magnetization is likely modern and can map the iron content of the crust, including possible local enhancements from impact-delivered iron. Strong magnetizations in a few places are puzzling: They may be a relic of an ancient stronger magnetic field, or reflect unusual magnetic properties of Mercury’s crust.

**Fig. 1.**
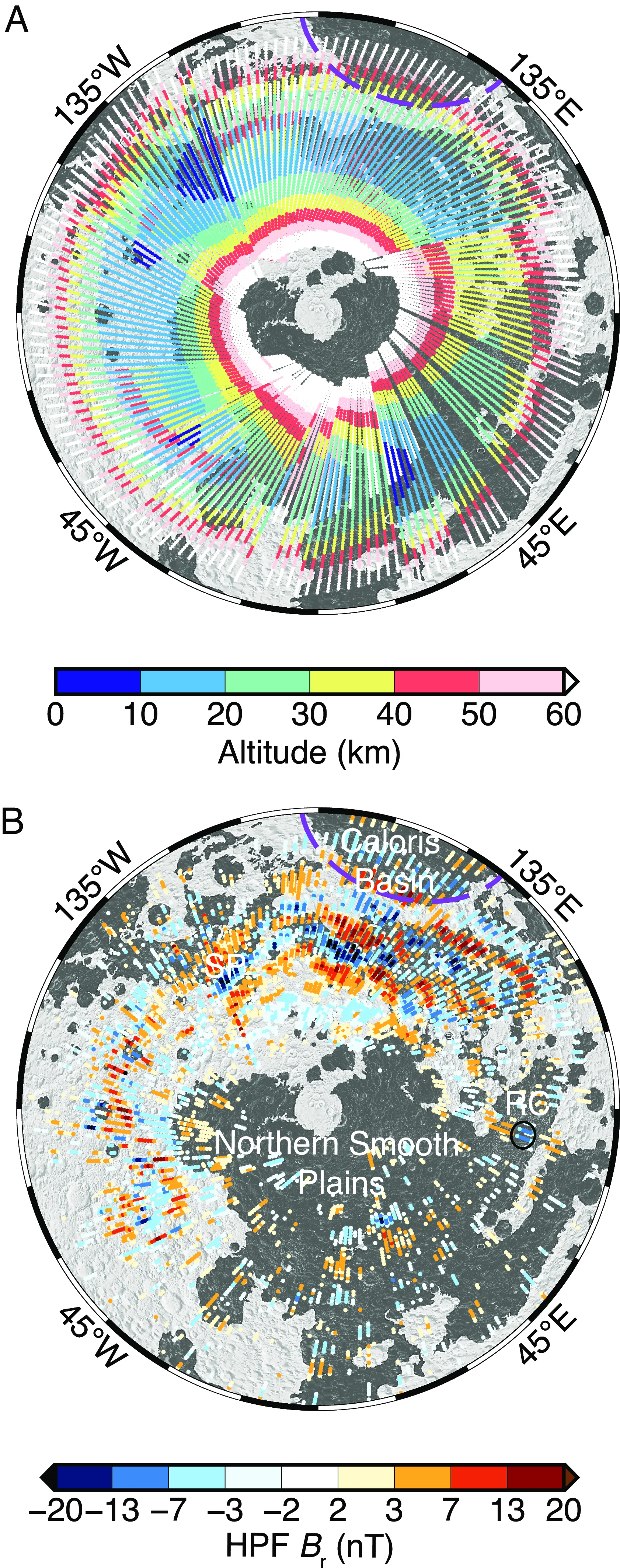
Distribution of MESSENGER crustal field observations. (*A*) Spacecraft altitude along orbit segments for which the spacecraft altitude was less than 80 km, for orbits with a magnetic disturbance index ≤ 95 (10). Background image shows smooth plains (dark gray) and intercrater plains (light gray), Lambert Azimuth equal area projection, 35 to 90 ^°^N. (*B*) Radial magnetic field component, $B_{r}$, in nT along the orbit segments in (A) after removal of noncrustal field sources with wavelengths > 800 km; $|B_{r}|>2$ nT (color), $|B_{r}|\leq 2$ nT not shown. The Northern Smooth Plains are labeled and the extent of the northern part of the Caloris basin is denoted by the purple dashed line. SP denotes Suisei Planitia (∼145 ^°^W, 60 ^°^N), the location of the original crustal field detections (9) and the black circle labeled RC denotes Rustaveli Crater (∼83 ^°^E, 52 ^°^N). MESSENGER 1 s data were subsampled by a factor of 6 for plotting.

Mercury’s global-scale internal field was first observed during the Mariner 10 flybys (1, 2) and was confirmed to be of core origin with data from the MErcury, Surface, Space ENvironment, GEochemistry and Ranging (MESSENGER) mission. The core field is dominantly dipolar, axisymmetric, and about 100× weaker than Earth’s field at the planetary surface (3–8). Crustal fields were discovered during the last few months of the MESSENGER mission (9) when spacecraft altitudes were less than ∼130 km (Fig. 1). The strongest crustal fields occur in and around the Caloris basin (9, 11, 12) (Fig. 1*B*) and at some, but not all, craters and basins (12).

Critical to understanding crustal fields is determining whether they result from remanent magnetizations indicative of a past dynamo, or from magnetizations induced in the present core field. An induced magnetization will have a direction and strength that is mainly determined by the present global field, but is also affected by immediately adjacent magnetizations (13). On Earth, crustal magnetization contains both induced and remanent contributions. Robust separation of these requires laboratory measurements of samples and is notoriously difficult with only satellite observations (14).

One approach to identifying remanent magnetization is to establish whether the magnetization direction is different from that of the present-day core field at the observation location. However, inversions of magnetic field data at satellite altitudes for magnetization models are nonunique (13, 15), so additional constraints are needed. For example, a constant magnetization direction is sometimes assumed, and an equivalent “paleopole” is calculated and compared with the present-day magnetic pole position (16). This model has been applied to five craters on Mercury (17): Four result in a wide range of paleopoles that include the present north magnetic pole position, with one feature suggesting a paleopole different from present day.

An alternative approach uses the strength of the observed crustal field, which is related to the volume-integrated magnetization. Magnetization strength is given by $M=\chi B/\mu_{0}$, where *B* is the field strength in which the magnetization is acquired, *χ* is the magnetic susceptibility and $\mu_{0}$ is the permeability of free space. The reduced nature of Mercury’s crust and mantle (18) means that iron metal or iron alloy minerals are the most likely magnetic minerals (9, 19). For an induced magnetiza tion, $B=B_{\mathrm{present}}$, and *χ* is the low-field susceptibility. For a remanent magnetization, $B=B_{\mathrm{ancient}}$, and *χ* is typically a thermoremanent susceptibility. Ref. 9 used this approach to conclude that the crustal fields over Suisei Planitia (Fig. 1*B*) are too strong to be induced in the present field, given the low iron content of Mercury’s crust (18). 12 reached the same conclusion for Rustaveli crater (Fig. 1*B*). A remanent magnetization was inferred, suggesting an ancient dynamo operating around the time of formation of the smooth plains at Suisei Planitia (∼3.8 Gyr ago) and the younger Mansurian-aged Rustaveli crater (∼3 to ∼1.7 Gyr old, 20). Furthermore, remanent magnetization can be thermally preserved for up to a few Gyr (9).

Although it has been shown that remanent magnetizations are possible, whether there are regions where the crustal field signals could be fully attributed to induced magnetizations has not been assessed. Establishing this is essential to understanding Mercury’s dynamo history and the distribution of magnetic minerals in the crust. If the magnetization is entirely induced, geographical variations in crustal magnetic fields reflect the abundance of magnetic carriers and/or lateral variations in magnetic mineral properties. If the magnetization is entirely remanent, crustal magnetic fields also record the dynamo history. If, as on Earth, both induced and remanent magnetizations are present, then teasing apart these contributions is critical to unraveling the planet’s paleofield history and could provide a novel way of probing lateral variations in the iron content and magnetic properties of the crust.

Here we use the complete low altitude dataset from the MESSENGER mission, to produce a magnetization model for Mercury’s northern latitudes to address the following fundamental outstanding questions: 1) What is the distribution and strength of magnetization? 2) How important are induced magnetizations? and 3) Can we deduce the planet’s dynamo history from the magnetization record?

## Data and Modeling Summary

The data processing steps and modeling approach are detailed in *Materials and Methods*; we give a brief summary below.

We use MESSENGER data at altitudes less than 130 km to isolate crustal magnetic fields at magnetospherically quiet times (Fig. 1 and *SI Appendix*, Figs. S1–S3 and Table S1). We retain signals with wavelengths less than 770 km, cf. 215 to 515 km in previous studies (*SI Appendix*, Table S1 and Fig. S4) (6, 9, 11, 12).

**Fig. 2.**
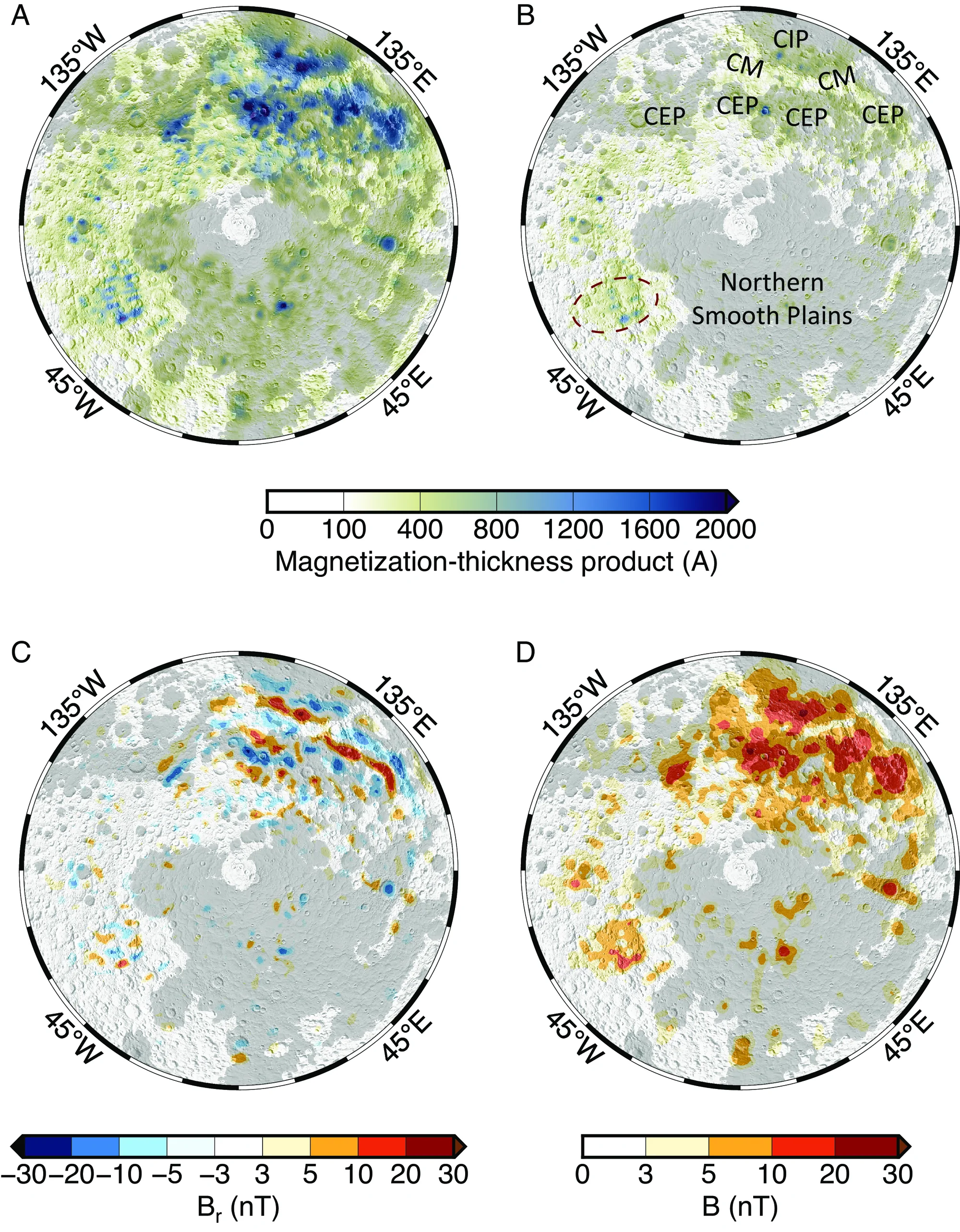
(*A*) Magnetization-thickness product and (*B*) Model uncertainty (*Materials and Methods*). Larger uncertainties, e.g. at ∼65 ^°^W (dashed brown oval) reflect data distribution rather than data quality, but even in these regions the signal-to-noise (model divided by the uncertainty) is greater than 1. (*C* and *D*) Predicted $B_{r}$ (*C*) and |B| (*D*) from the model in (*A*) at 20 km altitude. Figure projection as in Fig. 1, Smooth Plains units shaded darker gray. Major northern hemisphere physiographic features are labeled in panel (*B*). CIP and CEP denote the Smooth Plains units comprising the Caloris Interior Pains and Caloris Exterior Plains respectively. CM denotes the Intercrater Plains units comprising the Caloris Montes Formation that lie between the CIP and CEP. Note that Suisei Planitia (Fig. 1) is part of the CEP.

We invert for the magnetization-thickness product, *Mh* and its uncertainty at latitudes poleward of 32 ^°^N, using an equivalent source dipole (ESD) technique (21) (Fig. 2 and *SI Appendix*, Figs. S5–S7). The resulting *Mh* map is compared with the two major volcanic units on Mercury—the Intercrater Plains (ICP) and the Smooth Plains (SP), with major geochemical provinces and physiographic features, and with two end-member crustal thickness models—constant density model HGM008 (22) and V0, which has strong lateral variations in crustal density (23).

We estimate induced magnetizations from:

[1] $$\begin{array}{c} M_{\mathrm{ind}}=\chi w_{\mathrm{Fe}}B_{0}/\mu_{0}. \end{array}$$

$B_{0}$ is the present field strength, $w_{\mathrm{Fe}}$ is the crustal weight percent (wt%) iron and *χ* is the low-field susceptibility for the appropriate magnetic mineralogy(ies) containing 1 wt% iron. We assume that enstatite chondrites—extremely reduced, primitive stony meteorites that formed relatively close to the Sun—hold magnetic mineral assemblages that could plausibly be expected on Mercury and we use measurements from enstatite chondrites reported in table 5 of ref. 24 to constrain the average value of, and standard deviation in, *χ*. The average $w_{\mathrm{Fe}}$ for Mercury’s crust is ∼1.49 (18), but is quite different in the SP and ICP and, as described in *Materials and Methods*, we account for this in our calculations for $M_{\mathrm{ind}}$. We assume that $M_{\mathrm{ind}}$ is carried in a layer with thickness $h_{M_{\mathrm{ind}}}$ (the choice of value for $h_{M_{\mathrm{ind}}}$ is motivated by our inversion results, described later) and we compute $f_{1}$:

[2] $$\begin{array}{c} f_{1}=Mh/M_{\mathrm{ind}}h_{M_{\mathrm{ind}}}. \end{array}$$

$f_{1}$ allows us to identify regions where the crustal field can be fully explained by induced magnetizations, for nominal values for the inducing field strength, and the relevant magnetic properties of Mercury’s crust.

## Results

The largest *Mh* values occur in the Caloris region and as localized signals elsewhere (Fig. 2). Model amplitudes are well resolved as shown by the small uncertainties (Fig. 2*B* vs. *A*), even in areas of small *Mh* (Fig. 2). Overall, *Mh* shows no trend with latitude, i.e., the present dipolar core field structure is not reflected in the crustal magnetization. This is unsurprising even if the magnetization is induced; on Earth spatial variations in $M_{\mathrm{ind}}$ are controlled mainly by heterogeneities in the abundance and type of magnetic minerals and the magnetized layer thickness (14). The radial component and total field at 20 km altitude (Fig. 2*C*) reaches amplitudes of just over 20 nT. Predicted surface field strengths are up to 50 nT. Comparison with some previous models e.g., ref. 12, shows larger amplitudes here (*SI Appendix*, Fig. S8), as expected from the increased signal in the data used in the inversion (*SI Appendix*, Fig. S4). The crustal field model of ref. 25 for the Caloris region has a similar amplitude to our model.

### Correlations with Crustal Structure and Geochemical Provinces.

**Fig. 3.**
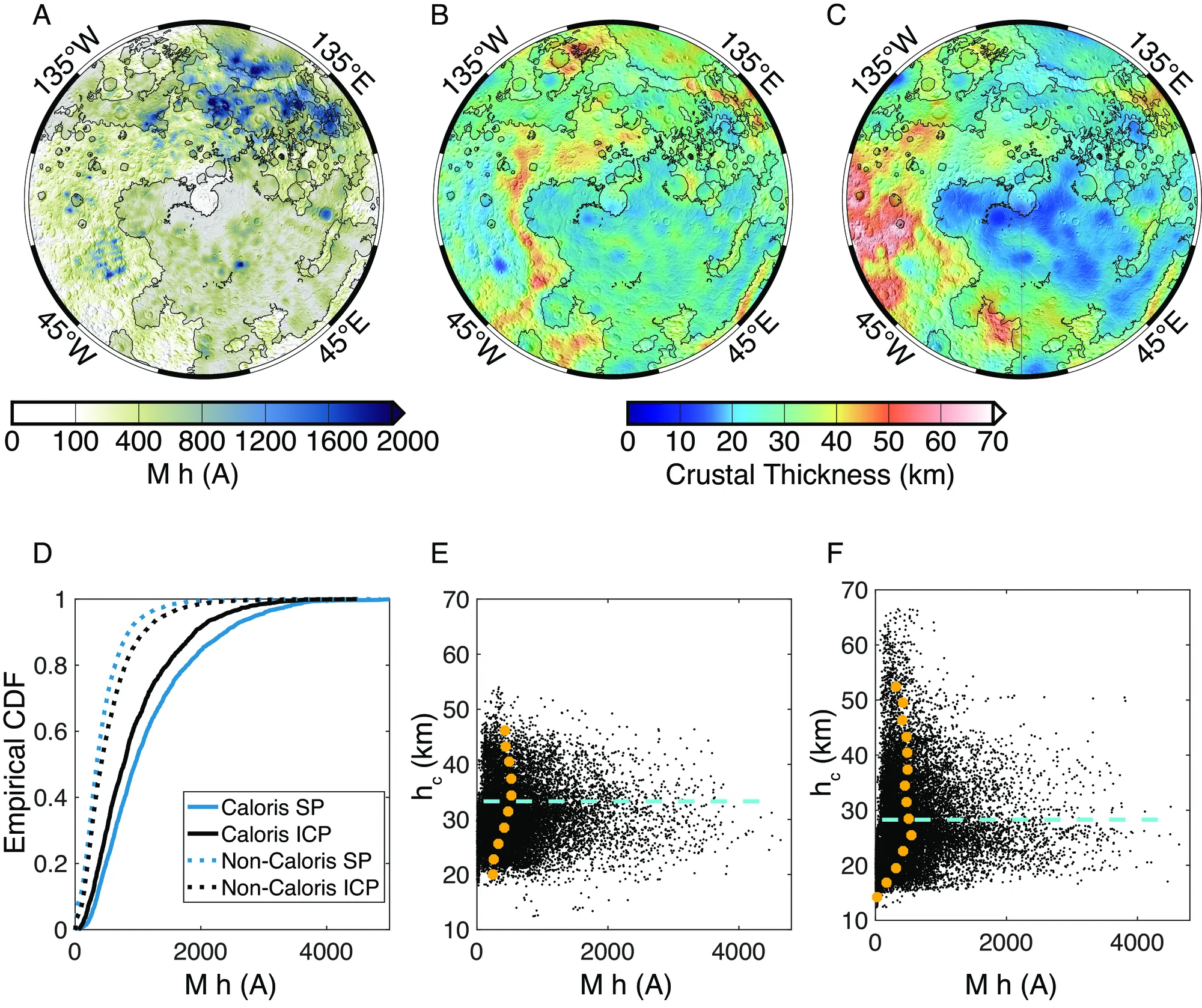
(*A*) Preferred model (as in Fig. 2*A*). (*B*) Crustal thickness model HgM0008 (22). (*C*) Crustal thickness model, V0 (23). (*D*) Empirical CDFs for the magnetization-thickness product (Mh) at locations with smooth plains (SP) or intercrater plains (ICP) units, in the Caloris region (longitudes 120 to 220 ^°^E and latitudes 35 to 70 ^°^N) and elsewhere. (*E*) HgM008 crustal thickness, $h_{c}$, vs. magnetization-thickness product, *Mh*, at equivalent source dipole (ESD) locations (black), with averages in 3 km crustal thickness bins (yellow asterisks). Dashed cyan line denotes the average crustal thickness, $\langle h_{c}\rangle =33.3$ km, north of 38 ^°^N at these ESD locations. (*F*) As in (*E*) for model V0, $\langle h_{c}\rangle =28.3$ km. For (*A*–*C*) Figure projection is as in Fig. 1, Smooth Plains units outlined in black.

We find a correlation of *Mh* with crustal thickness, $h_{C}$ for both crustal thickness models, HgM008 and V0, where $h_{C}$ is less than the average crustal thickness (∼28 km for V0 and 33 km for HgM008) (Fig. 3*A*–*C*, *E*, and *F*). For larger $h_{C}$, there is no correlation with *Mh*. This suggests that in regions where the crustal thickness is less than the average value, magnetizations could extend through much of the crustal column, and that the strongest *Mh* values occur independently of the thickest crust.

Outside the Caloris region the median *Mh* value over the ICP is 415 A c.f. 343 A over the SP (areally dominated by the Northern Smooth Plains, NSP) (Fig. 3 *A* and *D*), about a 20% difference. Thus the factor of 2.5 difference in crustal iron content inferred spectroscopically for the NSP (0.6 wt, 18) cf. the ICP (1.49 wt%, 18) is not reflected in the regional-scale crustal magnetization. The 20% difference in median *Mh* values could reflect bulk iron contents for the two geologic units that are more similar than implied by surface iron content. We find that a crustal column under the SP that comprises 72% ICP-like Fe content units and 28% SP-like Fe content units can explain the observed differences in *Mh* (*Materials and Methods*). Furthermore, this fractional contribution of ICP and SP units is also suggested by an independent approach using estimates for thickness of the SP units in large filled basins (26) (*Materials and Methods*).

Within the Caloris region, the largest *Mh* are associated with the SP units in the Caloris interior plains (CIP) and the Caloris exterior plains (CEP, sometimes referred to as the circum-Caloris plains). The spectrally red CIP are inferred to have low iron content, like the NSP, ∼0.6 wt% (18), whereas an iron content distinct from the global average value has not been reported for the spectrally blue CEP. We also find no correlations with distinct geochemical terrains; specifically the patchy stronger magnetizations at around 70 ^°^W, 60 ^°^N are northeast of the much larger high-magnesium region (mostly south of 55 ^°^N, at ∼60 ^°^W to 115 ^°^W) that is also inferred to have higher iron content (2.44 wt% Fe, 18).

### Induced Contributions and Excess Magnetizations.

**Fig. 4.**
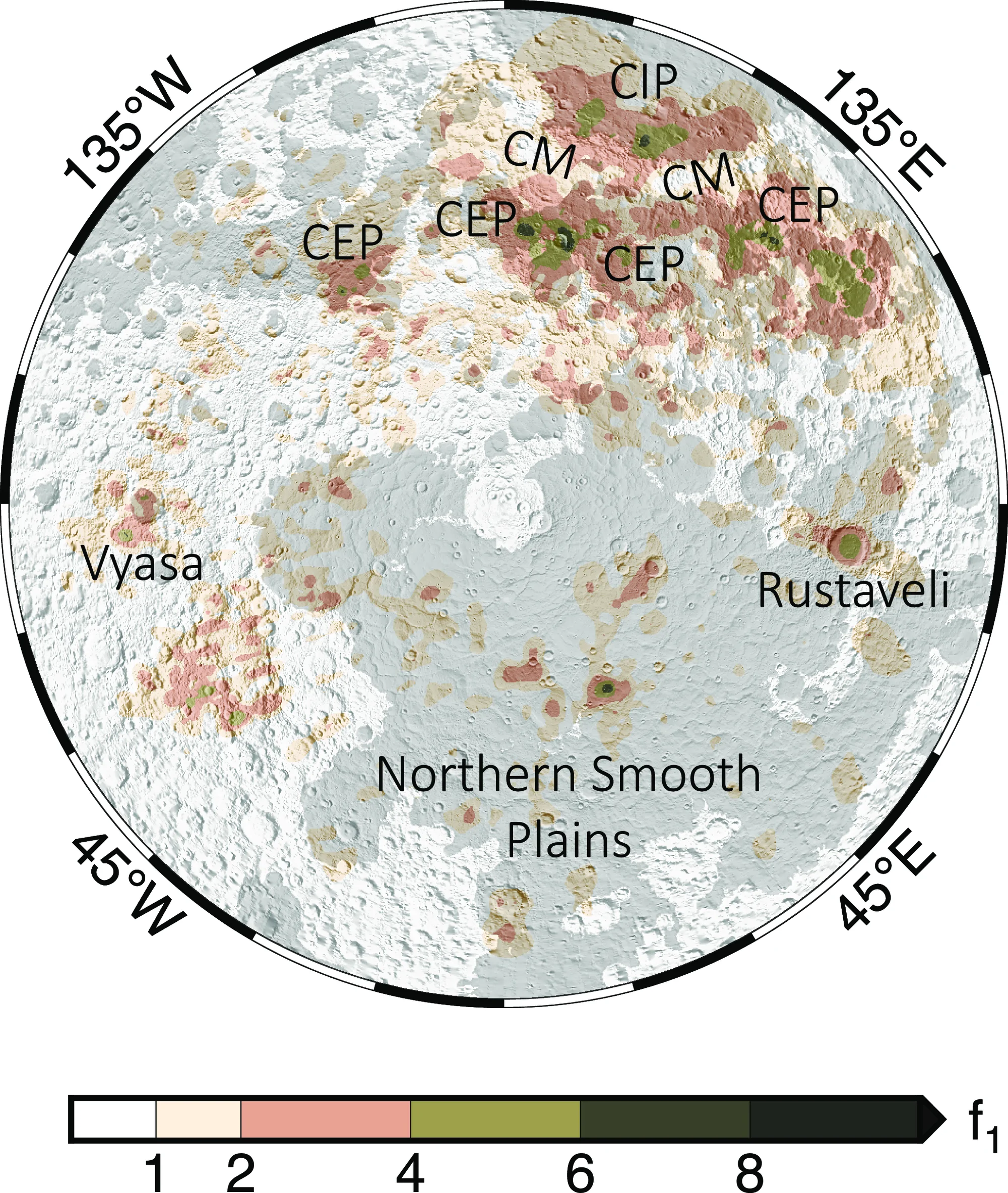
Factor, $f_{1}$, the ratio of the magnetization-thickness product to the magnetization induced in the present field for crustal thickness model HgM008 (Eqs. **1** and **2** and *Materials and Methods*). The magnetized layer thickness is assumed to be the crustal thickness, up to a maximum value of 30 km, and the bulk wt% Fe in the magnetized layer is assumed to be 1.49 wt% Fe for crust beneath the ICP and 1.23 wt% for crust beneath the SP (*Materials and Methods*). Smooth Plains shaded in darker gray. CEP, CIP, and CM denote the Caloris Exterior Plains, Caloris Interior Plains, and Caloris Montes ICP, respectively. Figure projection is as in Fig. 1.

To assess the contribution of induced magnetizations we compute $M_{\mathrm{ind}}$ (Eq. **1**) and use our results for the correlation of *Mh* with $h_{c}$ to set the induced magnetization layer thickness, $h_{M_{\mathrm{ind}}}=\max\left(h_{c},30\mathrm{km}\right)$. Induced magnetizations in Mercury’s crust are ∼0.030 A/m (*Materials and Methods*) and the corresponding $M_{\mathrm{ind}}h_{M_{\mathrm{ind}}}\approx$ 300 to 900 A for $h_{M_{\mathrm{ind}}}=10$ to 30 km. The resulting map for $f_{1}$ is shown in Fig. 4.

Crustal fields can be fully attributed to induced magnetizations in regions with values of $f_{1}$ (Eq. **2**) less than 1, and, for the upper 95% confidence limit on *χ*, in regions with $f_{1}<2$. $f_{1}$ is negligibly affected by the choice of crustal thickness model (*SI Appendix*, Fig. S9 cf. Fig. 4), and over the SP varies from ∼80% to ∼200% of that shown in Fig. 4 for end-member bulk iron contents beneath the SP units (*SI Appendix*, Fig. S10) and different choices of magnetization source depth (*SI Appendix*, Fig. S11). Thus induced magnetizations, although small, can account for 100% of the inferred magnetization-thickness product for 87% of the area north of 38 ^°^N. Furthermore, because the *Mh* model is well resolved everywhere, $f_{1}$ values and the area fraction with $f_{1}<2$ are not substantially affected by the model uncertainties (*SI Appendix*, Fig. S12).

**Fig. 5.**
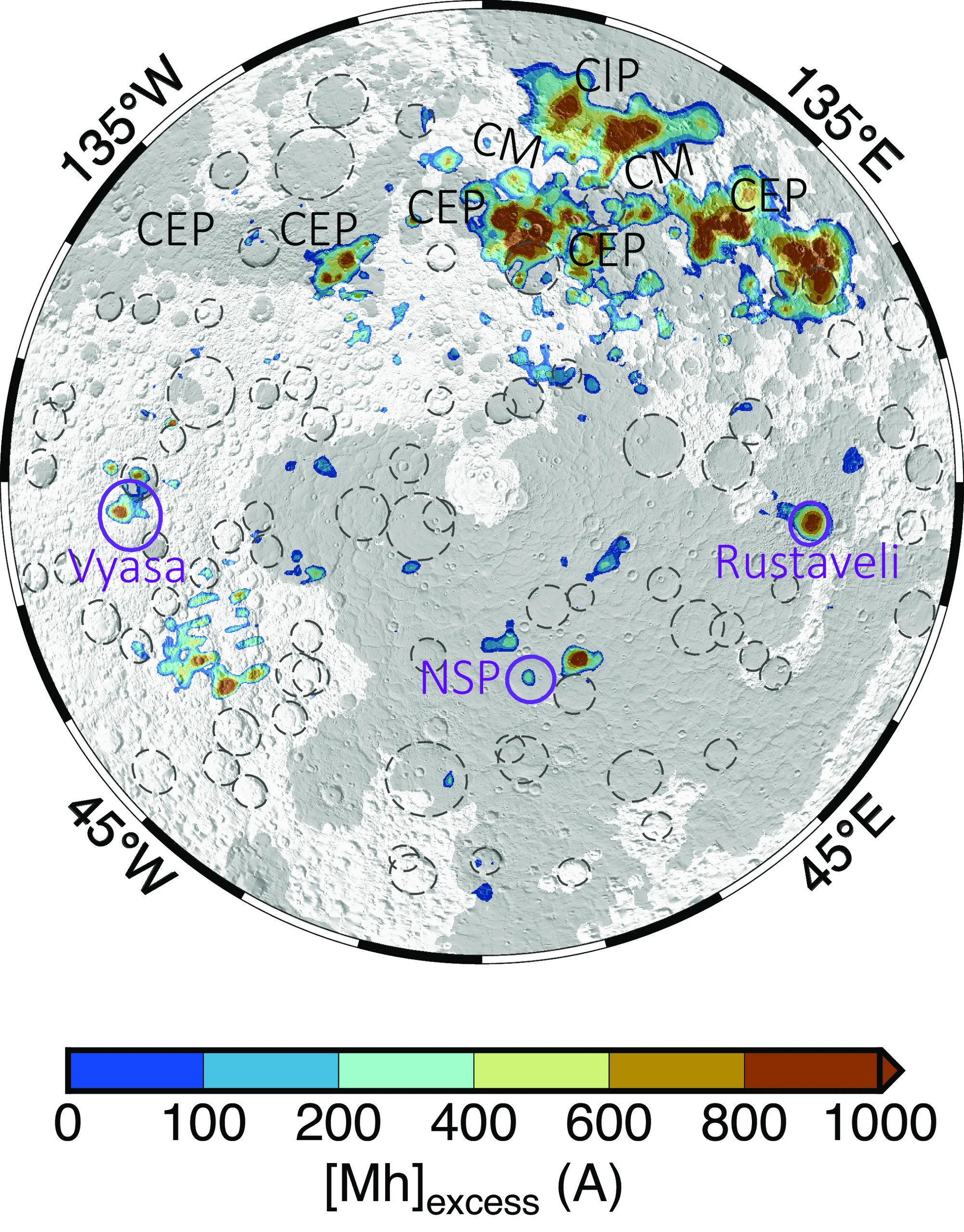
$Mh_{\mathrm{excess}}$, the magnetization thickness product in excess of that which could be induced in the present-field. Black dashed lines indicate the outlines of craters with diameters 125 to 400 km from ref. 27; three examples with $Mh_{\mathrm{excess}}>0$ are outlined in purple (Vyasa, Rustaveli, and “NSP”–a buried crater in the Northern Smooth Plains). Smooth Plains shaded in darker gray. CEP, CIP, and CM denote the Caloris Exterior Plains, Caloris Interior Plains, and Caloris Montes ICP, respectively. Figure projection is as in Fig. 1.

Where $f_{1}>2$ the crustal field strength implies either a larger bulk iron content for the crust than expected from spectroscopic observations, and/or a magnetizing field stronger than the present-day dipole field, and/or low-field susceptibilities outside the range of those published for enstatite chondrites (24). For these regions we compute ${\left[\mathrm{Mh}\right]}_{\mathrm{excess}}$, the magnetization-thickness in excess of that which could be induced (*Materials and Methods* and Fig. 5).

Regions with ${\left[Mh\right]}_{\mathrm{excess}}>$ 0 account for about 13% of the surface area north of 38 ^°^N (Fig. 5). For brevity we refer to these as strong magnetizations. Approximately 66% of the strongly magnetized area is associated with the Caloris region, bounded by longitudes 135 ^°^W, 120 ^°^E, and 70 ^°^N, including the CIP and CEP. About 60% of the remaining strongly magnetized area is associated with the SP and 40% with the ICP.

Our model offers improved constraints over previous work on the locations of the strongest signals in the Caloris region. 83% of the area with ${\left[Mh\right]}_{\mathrm{excess}}>0$ in the Caloris region is associated with the smooth plains in the CIP and CEP (Fig. 5). The remaining 17% is associated with some, but not all, of the Caloris Montes Formation ICP that lies between the CEP and CIP. The CEP and CIP account for 67% of the area in the region and the ICP 33%, i.e. the strongest signals are indeed concentrated in smooth plains units. Moreover, locally the areas in the ICP around Caloris with ${\left[Mh\right]}_{\mathrm{excess}}>0$ are typically immediately adjacent to smooth plains units.

## Discussion

Our model quantifies lateral variations in *Mh* on spatial scales of <∼800 km, and the results also provide indirect constraints on magnetization source depth. First, it is unlikely that the magnetization is concentrated in the upper few km of the crust, because there is no clear correlation of geographical variations in *Mh* with the inferred near-surface iron content. This interpretation is also consistent with previous work (6, 9, 25). Second, the results for *Mh* vs. crustal thickness suggest magnetic carriers are concentrated at depths less than 30 km. The lack of correlation of *Mh* with $h_{C}$ for $h_{C}>∼$30 km likely reflects the depth distribution of magnetic minerals rather than the effect of temperature on remanent or induced magnetizations. This is because i) remanent magnetizations are thermally stable throughout the crustal column for present-day or ancient thermal gradients (9), and ii) induced contributions are not expected to decrease markedly at any crustal depth, because the low-field susceptibility of iron or iron alloys is not strongly temperature-dependent at crustal temperatures (28).

As it is clear that induced magnetizations can explain observed crustal fields over much of the N. hemisphere, we next discuss regions with magnetizations that exceed the level expected from induced contributions. We examine whether these regions reveal clear information on a past dynamo and/or can be explained by larger induced signals that result from localized impact-delivered iron enhancements in the crust. We then discuss a major outstanding issue—namely whether measured low-field susceptibilities are representative of the magnetic properties of Mercury’s crust.

### Do Strong Magnetizations Reveal the Dynamo History?

A key issue is whether the regions with strong magnetizations (${\left[Mh\right]}_{\mathrm{excess}}>0$) can reveal Mercury’s dynamo history. We first consider the scenario in which all regions with ${\left[Mh\right]}_{\mathrm{excess}}>0$ require a contribution from remanent magnetization, and investigate whether these terrains are restricted to specific ages, relative or absolute.

In this scenario, the strong magnetizations in and around Caloris support suggestions that a core dynamo operated at the time of formation of the basin ∼3.94 Gyr ago (29) and the emplacement of the CIP and CEP (∼3.7 to 3.8 Gyr ago 9, 11, 29, 30, 31, 32). Furthermore, they require an ancient field stronger than the present field (9, 33). Previous work has interpreted crustal fields inside older (e.g. Vyasa and B31) and younger (e.g. Rustaveli) basins and craters to indicate a dynamo field at earlier and later times (11, 12). Exterior to the Caloris region, there are 77 craters with diameters 125 km to 400 km i.e., easily resolved in our magnetization model and for which a degradation class, a proxy for relative age, has been assigned. Of these, 14 (<20%) have strong magnetizations in their interiors, i.e. ${\left[Mh\right]}_{\mathrm{excess}}>0$. The degradation classes of these 14 craters span fresh (degradation class 4) to heavily degraded (degradation class 1) craters, with 4, 4, 1, and 2 craters of degradation classes 1, 2, 3, and 4 respectively, and an additional 3 craters that are buried beneath smooth plains units. Thus, if the interior magnetizations in these craters were acquired at the time the craters formed they reflect a dynamo present at ancient and more recent times.

However, the above inferences raise major questions: 1) If a dynamo existed at the time of formation of Caloris and the smooth plains in and around it, why are most of the smooth plains elsewhere not strongly magnetized? The weaker magnetization of the NSP c.f. the CIP is particularly puzzling given that the inferred wt% Fe of the NSP is indistinct from that of the CIP (18). 2) Why do so few craters record strong magnetizations if a dynamo was present throughout much of Mercury’s history?

### Are there Enhanced Local Induced Contributions from Impact-Delivered Iron?

Motivated by the above conundrum, we investigate the extent to which impact-delivered iron can lead to locally enhanced induced magnetizations, i.e. without requiring an ancient stronger field. Impact-delivered iron has been suggested previously to contribute to crustal fields over Rustaveli and Stieglitz craters (12, 34). Further, (12) concluded that impact-delivered iron cannot explain crustal field amplitudes over Rustaveli, assuming that the field can be modeled by iron from a 10 km impactor that is distributed in a spherical volume, radius 19 km, in the crust beneath the crater.

**Fig. 6.**
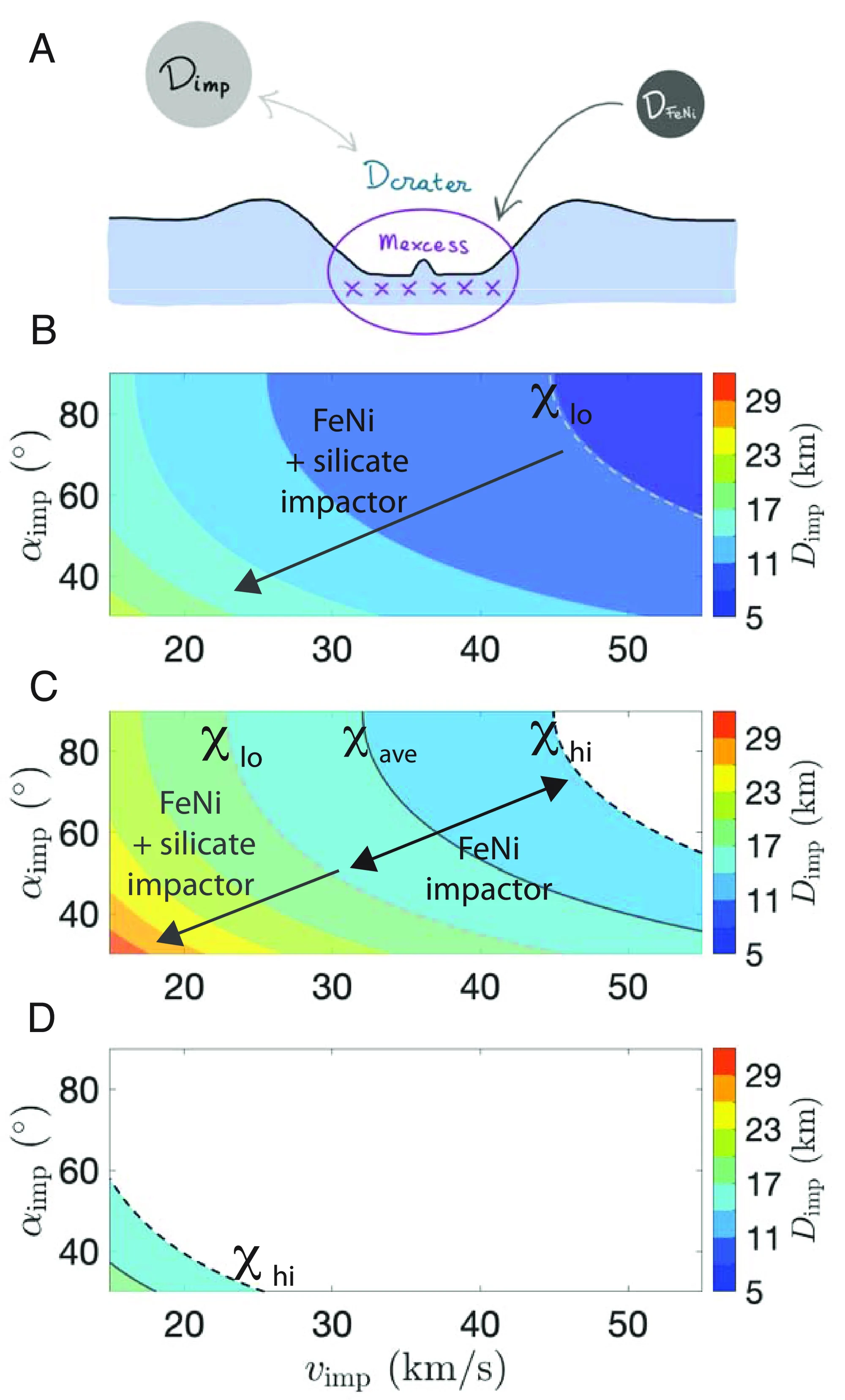
(*A*) Crater, diameter $D_{\mathrm{crater}}$, with interior excess magnetic moment $m_{\mathrm{excess}}$. Iron impactors, diameter $D_{\mathrm{imp}}$, can form $D_{\mathrm{crater}}$. $m_{\mathrm{excess}}$ can be generated by a volume of iron-nickel material, equivalent to a sphere with diameter $D_{\mathrm{FeNi}}$, that acquires an induced magnetization. If $m_{\mathrm{excess}}$ is fully explained by delivered iron, $D_{\mathrm{imp}}\geq D_{\mathrm{FeNi}}$. (*B*–*D*) Solutions for $D_{\mathrm{imp}}$ and $D_{\mathrm{FeNi}}$ for the three craters highlighted in Fig. 5—(*B*) NSP, (*C*) Vyasa, (*D*) Rustaveli. Black/gray curves show $D_{\mathrm{FeNi}}$ for the upper 1-*σ* (dark gray dashed, $\chi_{\mathrm{hi}}$), average (medium gray solid, $\chi_{\mathrm{ave}}$) and lower 1-*σ* (light gray dashed, $\chi_{\mathrm{lo}}$) susceptibility of iron meteorites. Filled colored contours show $D_{\mathrm{imp}}$ that produces $D_{\mathrm{crater}}$ for a range of impactor speeds $v_{\mathrm{imp}}$ and angles $\alpha_{\mathrm{imp}}$ ($\alpha_{\mathrm{imp}}=90^{{}^{\circ}}$ is a vertical impact). Highly oblique impacts, $\alpha_{\mathrm{imp}}<30^{{}^{\circ}}$ are not shown. White areas indicate ($v_{\mathrm{imp}}$, $\alpha_{\mathrm{imp}}$) conditions for which $D_{\mathrm{imp}}<D_{\mathrm{FeNi}}$, i.e. the impactor producing $D_{\mathrm{crater}}$ is too small to deliver sufficient iron to explain $m_{\mathrm{excess}}$. For $D_{\mathrm{imp}}$ values that exceed $D_{\mathrm{FeNi}}\left(\chi_{\mathrm{lo}}\right)$, $m_{\mathrm{excess}}$ and $D_{\mathrm{crater}}$ can be explained by impactors that need not be fully iron, as indicated in panels (*B* and *C*).

We use a different approach (*Materials and Methods*) that does not require assumptions regarding the distribution of delivered material inside the crater. We use the observed ${\left[Mh\right]}_{\mathrm{excess}}$ interior to the crater to compute the total excess magnetic moment inside the crater, $m_{\mathrm{excess}}$ and then calculate the diameter of an iron-nickel sphere $D_{\mathrm{FeNi}}$ that, if magnetized in the present field, can carry $m_{\mathrm{excess}}$. We use impact scaling laws to compute the diameter of an iron impactor, $D_{\mathrm{imp}}$, that can produce the observed crater diameter, $D_{\mathrm{crater}}$. For impact-delivered iron to fully explain the excess magnetic moment of the crater, $D_{\mathrm{FeNi}}$ cannot exceed $D_{\mathrm{imp}}$ (Fig. 6*A*, *Inset*). A systematic study of every crater is beyond the scope of this study but is underway (F. Cicchetti, personal communication, 2026). Here, we investigate three craters with clear interior observed ${\left[Mh\right]}_{\mathrm{excess}}$: Rustaveli (degradation class 4), Vyasa (degradation class 1), and a buried crater in the NSP (Figs. 5 and 6).

For the NSP buried crater, impact-delivered iron can explain the excess moment for any impact speed, angle, and Fe-Ni susceptibility (Fig. 6*B*). Moreover, because a chondritic impactor that can form $D_{\mathrm{crater}}$ will have a larger diameter than its iron impactor equivalent, the NSP crater $m_{\mathrm{excess}}$ does not require an iron-rich impactor. For example an impactor at 45^°^ and 25 km/s that comprises 33% by mass of iron could explain the magnetization enhancement within the crater. Similar conclusions hold for Vyasa (Fig. 6*C*), even though the basin itself is larger. $m_{\mathrm{excess}}$ can be explained by an iron-rich impactor for all impact conditions except very high speed, near-vertical impacts for the 1-*σ* bounds on Fe-Ni susceptibility and all impact conditions for the 2-*σ* bounds.

In contrast, at Rustaveli metal impactors can explain all of $m_{\mathrm{excess}}$ under only a limited range of conditions (Fig. 6*D*). Based on the symmetric distribution of ejecta materials at Rustaveli, (34) concluded that the impact angle must have been more than 40^°^. With this additional constraint impact speeds less than ∼25 km/s and Fe-Ni susceptibilities above the mean value are required to explain the observed $m_{\mathrm{excess}}$.

The extent to which impact ejecta materials are responsible for the strong magnetizations seen in and around Caloris remains an open question. MESSENGER altitudes mean that crustal fields were only measurable over the northern part of the basin and the CEP, areally comprising less than half of the Caloris region. Given this and the size of the Caloris basin, we do not attempt the calculations shown in Fig. 6 for Caloris, but simply note that even a chondritic impactor could have delivered a substantial amount of additional iron to the region. If the strong magnetizations are fully attributable to delivered iron, both the spatial and depth distribution of magnetization are puzzling. Why much of the enhanced magnetization is carried in the CEP and so little in the Caloris Montes ICP units is difficult to explain, as both are thought to contain Caloris impact ejecta (11), (32). Furthermore, magnetization source depths are estimated to be ∼10 km to ∼40 km for the region (25), arguing against impact-delivered materials unless these are mixed deep into the crust.

### Are Published Susceptibilities Representative of Mercury’s Crust?

Our results are based on the assumptions that published low-field magnetic susceptibilities are relevant in two ways. The first is whether low-field susceptibilities from enstatite chondrites, when scaled to Mercury’s inferred weight percent iron, are representative of the crust overall. This is important for establishing the spatial extent of regions with ${\left[Mh\right]}_{\mathrm{excess}}$, and the amplitude of ${\left[Mh\right]}_{\mathrm{excess}}$. The second is whether low-field susceptibilities from iron meteorites are appropriate for considering impact-delivered iron enhancements. We discuss each of these further below.

Susceptibilities have been measured on only a few enstatite chondrites (24) and show a small range in values (a factor of ∼4 for the 95% confidence limits on seven EH chondrites). Furthermore, because individual iron contents are not known for each meteorite, an average must be used to normalize the measured susceptibility values to 1 wt% iron (*Materials and Methods*). It is also possible that other magnetic carriers are present in Mercury’s crust that have not yet been magnetically characterized (19). As the crustal fields over more than 85% of the area north of 38 ^°^N can be attributed to induced magnetizations for current knowledge of *χ*, it is worth examining the susceptibilities that would be required for the crustal fields everywhere to be explained by induced magnetizations without a need for either remanent fields or localized impact-delivered iron enhancements. Even in the Caloris region, nominal background induced contributions contribute at least 50% of the observed signal over much of the region; and larger (smaller) susceptibilities would weaken (strengthen) the requirement for remanent magnetization, and in turn directly affect the strength of an ancient field needed to explain remanent signatures. Values for $f_{1}$ are less than 10 everywhere (Fig. 4), and $f_{1}=2$ corresponds to the upper 95% confidence limit on current estimates for *χ*. Thus the magnetizations could be fully explained as induced with an increase in *χ* of a factor of 5. If the average source depth for magnetization is ∼10 km (*SI Appendix*, Fig. S11*A*), rather than 20 km then an increase in *χ* of a factor of only 3 is required. For a scenario in which the bulk crustal susceptibility is increased relative to current knowledge, but to a value less than that required to attribute all crustal fields to induced magnetizations for the background wt% iron in the crust, ${\left[Mh\right]}_{\mathrm{excess}}$ is reduced in amplitude and spatial extent. Thus larger crater ${\left[Mh\right]}_{\mathrm{excess}}$ signals, such as at Rustaveli, can easily be explained by locally enhanced, impact-delivered iron.

In this paper we use susceptibilities from iron meteorites to investigate the possibility of enhanced induced magnetizations from impact-delivered iron. Iron meteorite susceptibilities show a large range (e.g. *SI Appendix*, Table S2 and Fig. 6). As expected, they are systematically larger than the lower limit of susceptibility of pure iron calculated based on theory (*Materials and Methods*) (24), but are notoriously challenging measurements to make. Even for unaltered meteorite samples (typically from meteorite falls) *χ* is difficult to measure using AC susceptibility meters because eddy currents are generated in the highly conductive iron meteorites by the rapidly oscillating magnetic field, resulting in spuriously low *χ* values. All of the measurements for meteorites reported by refs. 35 and 36 were collected using instruments that apply oscillating magnetic fields, and hence are likely to be lower than the true value. Although it is possible to try and correct for such eddy current effects (35), these corrections are rarely reported. However, a measured *χ* value for the Mungindi C iron meteorite was 3.1 (SI units), which after correction for eddy current effects and shape-induced demagnetization effects was 45 (35). This tenfold increase in susceptibility is close to a reported static DC value of 54.5 (37) on an unannealed 5 mm pure iron sphere. Annealed iron can have even higher values, investigated mostly via relative permeability in the materials science literature. A five-to-tenfold increase in iron meteorite susceptibilities cf. the average value used here, would mean that impact-delivered iron could easily lead to the enhanced magnetizations seen at some craters, even for cases like Rustaveli, without requiring a stronger ancient field. Eddy currents have not been discussed for enstatite chondrites, so we assume that the reported data are free of this effect. However these are also AC measurements (24), and if eddy currents are important even at a lower level than for iron meteorites, this could result in *Mh* everywhere being attributable to induced magnetizations, with or without local enhancements from delivered iron.

One final consideration in this discussion is that the *Mh* models here are likely lower bounds for two reasons. First, implicit regularization in the inversion produces models that have minimal amplitude at short spatial scales so that the models are stable to downward continuation. Second, some signal is missing in the crustal field observations at both the longest wavelengths (from the filtering needed to remove external fields) and the shortest wavelengths (observations very close to, or at, the surface would likely reveal additional signal, e.g., ref. 38). Whether these are significant effects for Mercury is unknown. If the true *Mh* is substantially stronger than that inferred this would, in turn, require greater iron contents and/or susceptibility if 100% of the magnetization is induced in the present field.

## Concluding Remarks

We conclude by returning to the three fundamental questions motivating our study:

### (1) What Is the Spatial Distribution and Strength of Magnetization Across Mercury’s Northern Latitudes?

Here we quantify Mercury’s crustal magnetization, via its magnetization-thickness product, *Mh* (Fig. 2). Strong magnetizations are associated with the Caloris region, in agreement with previous studies (9, 11, 12), and we find that large *Mh* are preferentially associated with the interior and exterior volcanic plains, rather than with the Caloris Montes formation immediately adjacent to the basin. Large-scale variations in near-surface iron content observed from MESSENGER spectroscopic data (18) are not reflected in *Mh* everywhere. For example, despite their similar inferred iron content (18), the Caloris Interior Plains and the Northern Smooth Plains have very different magnetic signatures. We find a correlation of *Mh* with crustal thickness, $h_{C}$, up to $h_{C}=30$ km (Fig. 3), suggesting that magnetic carriers are concentrated above this depth. Spatial correlations of crustal fields with crustal or lithospheric structure at scale lengths larger than 800 km (∼20^°^) are not possible because of the filtering required to isolate crustal fields. However, the strong magnetizations are more localized than the 800 km filter cut-off, and e.g., there are no suggestions of large-scale modulation of crustal fields with a pattern (spherical harmonic degree 3 and order 2) related to insolation-driven geographical variations in magnetization depth (39).

### (2) How Important are Induced Magnetizations?

Nominal values for induced magnetizations are small, as expected given the weak present-day core field (3) and the low iron content of Mercury’s crust (18). Even so, assuming that they are present throughout the crustal column up to depths of ∼30 km, induced magnetizations can fully account for the inferred *Mh* over more than 85% of the area north of 38 ^°^N (Fig. 4). Knowledge of the low-field susceptibility of Mercury’s crust as well as any currently undetectable crustal field signals are both key to understanding whether crustal fields everywhere can be attributed to induced signals. Relatively small increases in the background susceptibility will still leave areas for which currently measured crustal fields are unexplained by induced magnetizations, but that are easily explained with iron enhancements (of exogenic or endogenic origin) relative to the spectroscopically inferred values. Larger increases in the background susceptibility (a factor of ∼ 5) would predict induced magnetizations that could explain the MESSENGER-measured crustal fields everywhere without requiring remanent magnetizations or local iron enhancements. However, additional crustal field signals, currently undetectable by MESSENGER, would reduce the effects of larger low-field susceptibilities. For Mars, the InSight mission revealed that crustal magnetic fields at the planet’s surface can greatly exceed those measured at spacecraft altitude (38). Thus, Mercury’s crustal magnetic field and the corresponding $f_{1}$ (for the low-field susceptibilities currently in the published literature) may be greater than reported here.

### (3) Can We Deduce the Planet’s Dynamo History from the Magnetization Record?

Areas with magnetization-thickness products in excess of the nominal induced level (Fig. 5) are candidates for investigating Mercury’s dynamo history as the magnetization can comprise a remanent contribution. This presents puzzles, because not all terrains of similar ages require a remanent magnetization. Furthermore, enhanced induced magnetizations related to local impact-delivered iron enrichment may play a role in explaining this puzzle (Fig. 6). Given current understanding of the magnetic properties of Mercury’s crust (see below), the Caloris region and some craters point to the existence of a dynamo at 3.9 to 3.6 Gyr ago and at younger times, including the time of the Rustaveli impact; however, even modest increases in the background induced level could negate the requirement for remanent magnetizations as discussed above.

#### Looking ahead.

Progress in understanding Mercury’s crustal field requires advances in several areas.

First, conclusions regarding induced magnetizations are critically dependent on the low-field susceptibility of the crust inferred from meteorites or analog mineralogies. Existing laboratory measurements are limited in number and for high iron content meteorites challenged by technical issues. For iron meteorites in particular these may be underestimates of the true susceptibility. Progress in addressing this issue is paramount for identifying whether there is any requirement for remanent magnetization, and hence constraints on an ancient field.

Second, Bepi-Colombo data will contribute substantively to understanding the MESSENGER record of crustal fields in a variety of ways. Better core field models (40) will enable more accurate induced magnetization calculations. Improved characterization of crustal structure (41) and of surface composition, especially increased resolution in iron maps, will be important for interpretation of magnetization models.

Finally, current Bepi-Colombo mission plans (42) indicate both spacecraft will be above 250 km altitude until 2029, and thus crustal fields will only be measured if they are very different in character from even the strongest fields seen to date over Caloris. However, both spacecraft will eventually fly at altitudes <100 km, enabling new detection of crustal fields at low and southern hemisphere latitudes.

## Materials and Methods

### Data Selection and Processing.

We use 1 s averaged vector magnetic-field data (43) taken at altitudes less than 130 km (Fig. 1*A*). The data are dominated by core and magnetospheric fields, and the radial field component, $B_{r}$, reaches ∼600 nT amplitude (*SI Appendix*, Fig. S1*A*). To isolate crustal fields we proceeded as follows (see also refs. 6 and 9).

We applied three criteria to minimize external field contamination. First, we exclude orbits affected by Interplanetary Coronal Mass Ejections (44), or for which the magnetospheric disturbance index (10, 45) exceeded 95. Second, we use only $B_{r}$ in our modeling, because the horizontal components are more affected by external fields (*SI Appendix*, Fig. S2). Third, we excluded data taken in the cusp (local times 3:40 pm to 7:20 am). At altitudes >∼ 80 km altitude we impose more stringent selection criteria, retaining only data at latitudes less than 70 ^°^N and with local times between 6 pm and 6 am (*SI Appendix*, Fig. S3).

We subtract from the data the predictions of a model that includes the offset axial dipole, magnetopause, and magnetotail fields (46). The residual signals have amplitudes of tens of nT (*SI Appendix*, Fig. S1*B*), are organized in the local time frame and vary with magnetospheric activity, indicative of an external origin (9, 47–49).

We use a high-pass filter (HPF) along-track to remove these remaining long-wavelength external fields (Fig. 1*B*), and retain signals with wavelengths less than 770 km (Fig. 1*B*). Previous work (9) retained signals with wavelengths less than 515 km or even just 215 km (11, 12). The less restrictive HPF used here was also found to be an acceptable choice (see *SI Appendix*, Fig. S5 of Johnson et al. 9). This is consistent with the mapped spatial extent of Birkeland currents that are dominated by signals with wavelengths ≥∼800 km (47, 48), and with previous filtering required to isolate regional core fields (49). This filtering removes any fields with spatial scales larger than 800 km, regardless of their origin. We also implement a HPF that retains data with wavelengths <215 km for comparison of our data with those used in refs. 11 and 12 (*SI Appendix*, Table S1 and Fig. S4). Many regions devoid of signals in the more heavily filtered dataset, now show weak signals and amplitudes are increased overall.

### Inversions.

Following refs. 25 and 49, we randomly subsampled the HPF data with respect to their altitude and along-track vs. cross-track spacing to obtain a hundred “modeling” data subsets together with their complementary “evaluation” subsets, each with a more equal-area-distribution compared to the full dataset. Each of these hundred modeling subsets were inverted using an ESD inversion (21), with an iterative conjugate gradient solver. Our inversions used a single equivalent source layer and a dipole spacing of 25 km, appropriate for both the along-track and cross-track resolution. 98.5% of the HPF data below 130 km altitude are north of 35 ^°^N and 99.7% are north of 32 ^°^N. The modeled region is thus 32 ^°^N to the pole, and to model this without edge effects a 10^°^ latitude buffer is included in the inversion, i.e. the inverted region extends to 22 ^°^N. The resolvable model features are north of 32 to 35 ^°^N as seen in *SI Appendix*, Fig. S5*B* and as expected based on the latitudinal distribution of the low altitude data.

The ESD approach solves for the moment ($\vec{m}$) of each source dipole which is related to the magnetization, $\vec{M}$, by $\vec{m}=\vec{M}V$, where *V* is the volume associated with each source dipole. As the area associated with each dipole spacing is known for a given dipole spacing, the quantity estimated is the magnetization thickness product, $\vec{M}h$, which we discuss in terms of its amplitude *Mh*. As the inversion proceeds, models increase in complexity and provide a lower (better) misfit to the modeled data globally (*SI Appendix*, Fig. S5 *A*–*C* and *G*) and regionally (*SI Appendix*, Fig. S5 *D*–*F* and *H*) and to the evaluation data (*SI Appendix*, Fig. S5*G*). However as the model complexity increases, structure warranted by a given modeled data subset may not be required by the corresponding evaluation data, resulting in an increase in the misfit to the evaluation data (*SI Appendix*, Fig. S5*G*).

The amplitude of our resulting model is important because this quantity is compared with possible induced contributions. Factors affecting model amplitude that result from the inversion choices are a) the choice of final model on the trade-off curve, b) source depth, and c) whether the magnetization direction is constrained. We discuss each of these below.

We chose the preferred model from the iteration in the average trade-off curve that has the minimum misfit to the evaluation subsets (Fig. 2*A* and *SI Appendix*, Fig. S5*G* red dot). The preferred model (Fig. 2*B* and *SI Appendix*, Fig. S5) is evaluated from the average of the bootstrap models at this iteration, where the average is computed for each component of the ESD moments, and the model magnitude is computed from the norm of the average components. As *Mh* is not normally distributed we compute the uncertainty, $s_{Mh}$ in *Mh* from half the difference between the 17th and 83rd percentile models, i.e. $\pm 1s_{\mathrm{Mh}}$ is analogous to ±1σ for a normally distributed variable. The resulting model has the minimal structure and amplitude required to explain both the modeled and evaluation data subsets. The model amplitude everywhere is larger than the model uncertainty. Larger model uncertainties are a natural consequence of the bootstrap inversion in regions that lack duplicate coverage. This is the case for the region at ∼65 ^°^W (Fig. 2*B*, dashed brown circle): The larger uncertainties reflect data distribution, rather than inherently lower quality observations.

We investigated the dependence of model amplitude on source depth (*SI Appendix*, Fig. S6) $Z_{S}$, for $Z_{S}=10$ km to 40 km below Mercury’s average radius, i.e., ∼5 km to ∼35 km below the lowest elevations. This can be thought of as the depth to the middle of the magnetized layer, thus a choice of $Z_{S}=6$ km constrains the maximum magnetized layer thickness to be ∼12 km. Deeper sources require larger model amplitudes, but the overall spatial structure is similar (*SI Appendix*, Fig. S6). The variation in the RMS model amplitude for crustal source depths is a factor of ∼2.

Models with different magnetization directions can fit the data equally well (13, 15). We inverted for both an unconstrained dipole direction (Fig. 2) and a dipole direction constrained to be in the direction of the present field (*SI Appendix*, Fig. S7). These yield models with similar overall structure, but in the case of the constrained direction, all structure in the data has to be absorbed by lateral variations in amplitude of the magnetization (*SI Appendix*, Fig. S7*A* vs. Fig. 2*A*), resulting in a larger model norm. The point-wise ratio of the amplitudes of the models in *SI Appendix*, Fig. S7*A* and Fig. 2 has a modal value of 2. An unconstrained direction is typically used for inversions for remanent magnetization (e.g., refs. 21 and 50) as the ancient magnetizing field and resulting magnetization need not be aligned with the present field. In theory, if the magnetization is entirely induced, it will be aligned with the present field, but in practice magnetizations are also affected by the local magnetic field of neighboring magnetized rocks. At Mercury there may be both remanent and induced contributions, thus we use the results from the unconstrained direction and take differences in model amplitudes into consideration in our calculations of induced contributions.

### Induced Magnetizations.

The magnitude of, and uncertainty in, $M_{\mathrm{ind}}$ depend on $B_{0}$, *χ*, and $w_{\mathrm{Fe}}$, (Eq. **1**). $B_{0}$ varies from ∼300 nT to 700 nT over our modeled latitudes. Motivated by our inversions which show no correlation of *Mh* with latitude, we use a single value for $B_{0}$ of 560 nT, corresponding to the strength of the internal field at the surface at 60 ^°^N (i.e., close to periapsis). We compute an average value and 1-*σ* and 2-*σ* confidence limits on *χ* from laboratory measurements on EH chondrites (24), first converting the reported values in table 5 of ref. 24 to SI units using the mean density for EH chondrites (3,720 kg m^−3^) and then normalizing by 25 wt% Fe, the average weight per cent iron that is attributable to nonsulfide magnetic carriers (51, 52). This yields χ=0.045 (SI units) with lower and upper limits a factor of ∼ 2 smaller and larger respectively. For $w_{\mathrm{Fe}}$, we treat the ICP and SP separately. We use the global average of 1.49 wt% Fe for the ICP. Beneath the SP, it is unknown how much of the crustal column comprises buried ICP units. We use two independent approaches to estimate this. First, as two end members, the observed 20% difference in the median *Mh* for the SP and ICP units outside Caloris could reflect a 20% difference in the thickness *h* of the magnetized layer in the ICP vs. the SP if they have the same bulk iron content and/or 20% difference in the bulk iron content of the crustal column if the magnetized layer thicknesses are on average similar. We take the second scenario as more likely given that there are observed differences in surface iron content (53) and that differences in crustal thickness beneath the ICP and SP are strongly dependent on assumptions about lateral variations in density (22, 23). A 20% difference in bulk iron content is achieved if the crust beneath the SP comprises 28% by thickness SP units with 0.6 wt% Fe and 72% ICP units with 1.5 wt% Fe. Second, buried craters in the NSP provide a lower limit on the thickness of the more recent SP units of ∼200 m to ∼2 km (54–56). (26) propose much larger NSP thicknesses based on depths of 10.1 km, 7.4 km, 6.5 km, and 7.8 km for four larger, filled basins. We use the average of these estimates, 8.1 km, for the thickness of the SP, which corresponds to 29% of the average crustal thickness in the NSP for crustal thickness model HgM008 (22), consistent with the 28% inferred from the first approach. Thus for our nominal induced contribution we calculate $w_{\mathrm{Fe}}$ beneath SP units from the weighted average, $w_{\mathrm{Fe}}=0.29*0.6+0.71*1.49=1.23$.

$f_{1}$ (Eq. **2**, main text) compares the inferred *Mh* with that expected from an induced magnetization. To compute $f_{1}$ we use the model in Fig. 2, scaling the model amplitudes by a factor of 2, to account for the larger model norm in *SI Appendix*, Fig. S7 cf Fig. 2. Both *Mh* and $h_{M_{\mathrm{ind}}}$ vary spatially, so $f_{1}$ also varies spatially. We explore how $f_{1}$ is affected by i) different crustal thickness models (*SI Appendix*, Fig. S9), ii) end member choices of 0% and 100% for the contributions of the lower weight per cent iron SP units to the crustal column beneath the SP (*SI Appendix*, Fig. S10), iii) the influence of source depth (*SI Appendix*, Fig. S11), and iv) consideration of the uncertainty in our *Mh* model (*SI Appendix*, Fig. S12). For the latter calculation we compute $f_{1}$ for the 17th percentile model for *Mh* (i.e. the average model (Fig. 2*A*) minus the model uncertainty (Fig. 2*B*)).

### Excess (“Strong”) Magnetizations.

For the upper 95% confidence limit on *χ* the factor $f_{1}<2$ corresponds to regions that can be fully attributed to induced magnetizations. For regions with $f_{1}>2$ we compute the magnetization-thickness in excess of that which could be induced for the upper 95% confidence limit on *χ*, i.e., ${\mathrm{Mh}}_{\mathrm{excess}}=\mathrm{Mh}-2M_{\mathrm{ind}}h_{M_{\mathrm{ind}}}$. Regions and craters (27) with ${\mathrm{Mh}}_{\mathrm{excess}}>0$ were assessed by determining whether at least two dipole points with ${\mathrm{Mh}}_{\mathrm{excess}}>0$ lay within the specified region or crater. We identified craters exterior to Caloris region as those with centers outside the edge of the CEP. A systematic study of crater magnetizations is beyond the scope of this paper but is underway (F. Cicchetti, personal communication and https://doi.org/10.5194/egusphere-egu26-14037, https://www.hou.usra.edu/meetings/lpsc2025/pdf/1993.pdf).

### Impact-Delivered Iron.

For each crater we compute the observed excess magnetic moment, $m_{\mathrm{excess}}=\Sigma_{i=1}^{N}{\left[Mh\right]}_{{\mathrm{excess}}_{i}}\Delta A_{i}$, where the sum is over the *N* model dipoles inside the crater each representing an area $\Delta A_{i}$.

We assume that impact-delivered iron is likely to be concentrated in the upper crust (0 to ∼20 km depth), so we adjust $m_{\mathrm{excess}}$ computed from the 20 km source depth model in Fig. 6 to a source depth of 10 km using the factor of 0.8 in *SI Appendix*, Fig. S7*D*. We calculate the diameter of an iron-nickel sphere, $D_{\mathrm{FeNi}}$ that has a magnetic moment, $m_{\mathrm{excess}}$, if magnetized in the present field, $B_{0}$:

[3] $$\begin{array}{c} D_{\mathrm{FeNi}}={\left(\frac{3\mu_{0}m_{\mathrm{excess}}}{4\pi \chi_{\mathrm{FeNi}}B_{0}}\right)}^{\left(1/3\right)}, \end{array}$$

where $\chi_{\mathrm{FeNi}}$ is the low field susceptibility of the iron-nickel sphere. We determine a value for $\chi_{\mathrm{FeNi}}$ from measurements on 12 iron meteorites (*SI Appendix*, Table S2). These have a mean density of 7,571 kg m^−3^ and a mean *χ* of 1,060 × 10^−6^ m^3^ kg^−1^, yielding a dimensionless value for $\chi_{\mathrm{FeNi}}$ for Eq. **3** of 8.0, with 95% confidence limits 2.6 and 24.7. We note that the value of 3.0 used by ref. 12 is a theoretical lower limit for pure Fe-Ni (24). This is lower than the average for iron meteorites but within the 95% confidence limits. We compute a range of $D_{\mathrm{FeNi}}$ using the 95% confidence limits for $\chi_{\mathrm{FeNi}}$. Importantly, this approach requires no assumption about how the delivered iron is distributed within the crater.

We use Π-scaling assuming cratering in a gravity-dominated regime and impacts into competent rock to calculate the transient crater diameter $D_{\mathrm{tr}}$, for given impact conditions (57). From ref. 57, $\Pi_{D}=C_{D}\Pi_{2}^{-\beta }$, where $\Pi_{D}=D_{\mathrm{tr}}{\left(\rho_{\mathrm{target}}/m_{\mathrm{imp}}\right)}^{1/3}$, $\Pi_{2}=1.61gD_{\mathrm{imp}}/v_{\mathrm{imp}}^{2}$, where $D_{\mathrm{imp}}$, $m_{\mathrm{imp}}$, $v_{\mathrm{imp}}$ are the impactor diameter, mass, and speed, respectively, $\rho_{\mathrm{target}}$ is the target density and $C_{D}=1.6$ and β=0.22. We also assume the relationship between the transient crater diameter and the observed crater diameter to be $D_{\mathrm{tr}}=D_{\mathrm{sc}}^{0.15}D_{\mathrm{crater}}^{0.85}$ (58), where $D_{\mathrm{sc}}$ is the simple-to-complex transition crater diameter, $D_{\mathrm{sc}}=11.7$ km (59). To calculate the diameter of an impactor that can produce the observed crater diameter $D_{\mathrm{crater}}$ (27) for each of three example craters (Fig. 6): Rustaveli (191 km), Vyasa (310 km), and a buried crater in the NSP (220 km), the combined equation is

[4] $$\begin{array}{c} D_{\mathrm{imp}}={\left[\frac{D_{\mathrm{sc}}^{0.15}\;D_{\mathrm{crater}}^{0.85}}{1.16\;{\left(\rho_{\mathrm{imp}}/\rho_{\mathrm{target}}\right)}^{1/3}\;{\left(v_{\mathrm{imp}}\;\sin\alpha \right)}^{0.44}\;g_{\mathrm{target}}^{-0.22}}\right]}^{1/0.78} \end{array}$$

$D_{\mathrm{imp}}$ depends on the target properties: density, $\rho_{\mathrm{target}}$ (3,400 kg m^−3^ for Mercury’s crust), gravitational acceleration, *g* (3.7 ms^−2^), and $D_{\mathrm{sc}}$. We assume an iron impactor, density $\rho_{\mathrm{imp}}=7,800$ kg m^−3^, and compute $D_{\mathrm{imp}}$ for all impact angles, *α*, and for a range of impact speeds, $v_{\mathrm{imp}}=15$ to 55 km s^−1^, applicable to Mercury (60). Impact-delivered iron can explain the excess magnetization in a crater if $D_{\mathrm{FeNi}}\leq D_{\mathrm{imp}}$. The condition $D_{\mathrm{FeNi}}\leq D_{\mathrm{imp}}$ vs. $D_{\mathrm{FeNi}}<<D_{\mathrm{imp}}$, requires that there are at least some impact conditions for which an iron impactor can be fully or mostly retained. Literature suggests that iron is easier to retain than rock, due to the much higher pressures required for melting and vaporization of iron (61). Recent numerical shock physics calculations indicate that this is indeed the case for iron impactors with speeds less than the median speed and diameters that could produce the example craters shown in Fig. 6 (K. Miljković, personal communication, December 2025 and accepted abstract PS11-003 AOGS 2026). The diameters computed using the scaling law above are different from those reported by ref. 12 by almost a factor of 2. The latter were obtained using an online calculator and at the time of original submission of this manuscript we used that online calculator (https://www.eaps.purdue.edu/impactcrater/) to verify the reported numbers in ref. 12. The reason for the difference is unclear and unfortunately at the time of submission of this revised manuscript the online calculator has now been modified to run for only Earth impacts. We use the values obtained from the easily reproducible Eq. **4** (57), which was also used for Mercury by ref. 62. Eq. **4** above corrects two typographical errors in equation 2 of ref. 62—the density ratio ($\rho_{\mathrm{imp}}/\rho_{\mathrm{target}}$ not $\rho_{\mathrm{target}}/\rho_{\mathrm{imp}}$) and the exponent for impact speeds. Furthermore, recent numerical models addressing impact scaling relationship for impact basins on Mars (63) suggested that gravity (which is similar for Mars and Mercury) is the dominant factor in impact scaling up to the 400 km basin diameters considered here.

## Supplementary Material

Appendix 01 (PDF)

## Data Availability

Files containing the high-pass filtered data (Fig. 1), the equivalent source dipole model and predicted crustal magnetic field (Fig. 2) are available from ref. 64. Derived products (Figs. 4 and 5) were generated using the workflow provided in *Materials and Methods*. Other datasets/models used here e.g. the crustal thickness models, V0 and HGM008, are available from the cited publications. The high-pass filtered data file was generated from 1s vector magnetic field data archived at the PDS, and the file archived on Zenodo includes time stamps and the prefiltered data to allow cross-referencing of these files. All other data are included in the manuscript and/or *SI Appendix*.
