# Supplementary material for: The distribution and origin of Mercury’s crustal magnetization
Source: Proc Natl Acad Sci U S A. 2026 Aug 17;123(35):e2535723123. doi: 10.1073/pnas.2535723123 (PMC13535193; doi:10.1073/pnas.2535723123)
Supplement: Supplementary file 1 [file pnas.2535723123.sapp.pdf]

Supporting Information for

The Distribution and Origin of Mercury's Lithospheric Magnetization

Catherine L. Johnson, Alain M. Plattner, Filippo Cicchetti, Katarina Miljković

Catherine L. Johnson
E-mail: cjohnson@eoas.ubc.ca

This PDF file includes:

Figs. S1 to S12
Tables S1 to S2
SI References

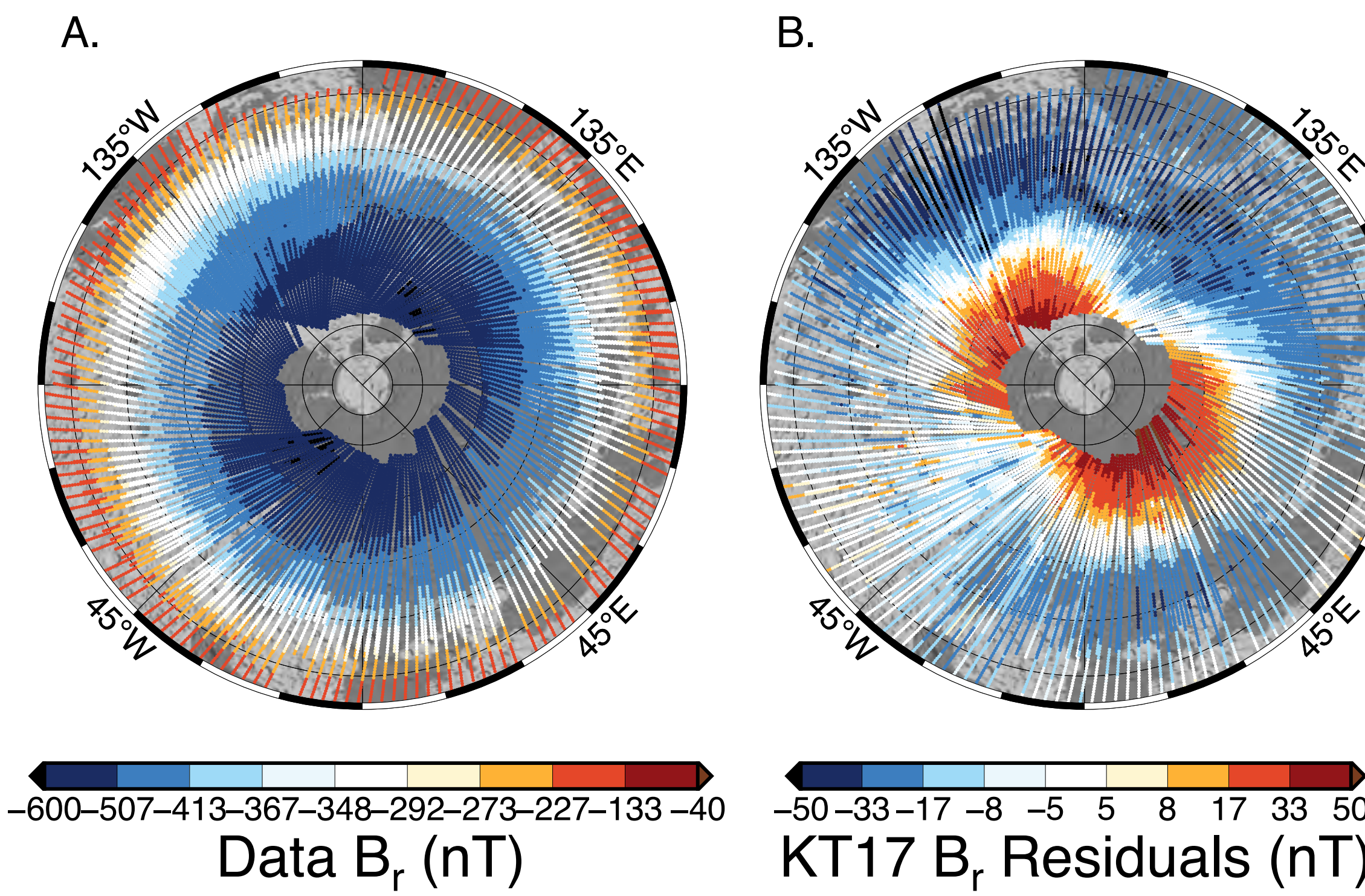

Fig. S1. (A) Radial magnetic field component, B_r, in nT along the orbit segments shown in Fig 1A of main paper shown in Mercury Body Fixed coordinates. (B) Residuals after subtraction of the magnetospheric model of (1) from the data in (A). Most of the remaining long-wavelength structure is of external origin (2). High-pass-filtering of the residuals results in the data shown in Fig. 1B of main paper. MESSENGER data were subsampled by a factor of 6 for plotting. Background images shows smooth plains (dark gray) and intercrater plains (light gray), grid lines every 30° in longitude and every 10° latitude, Lambert Azimuth equal area projection, 35–90 °N.

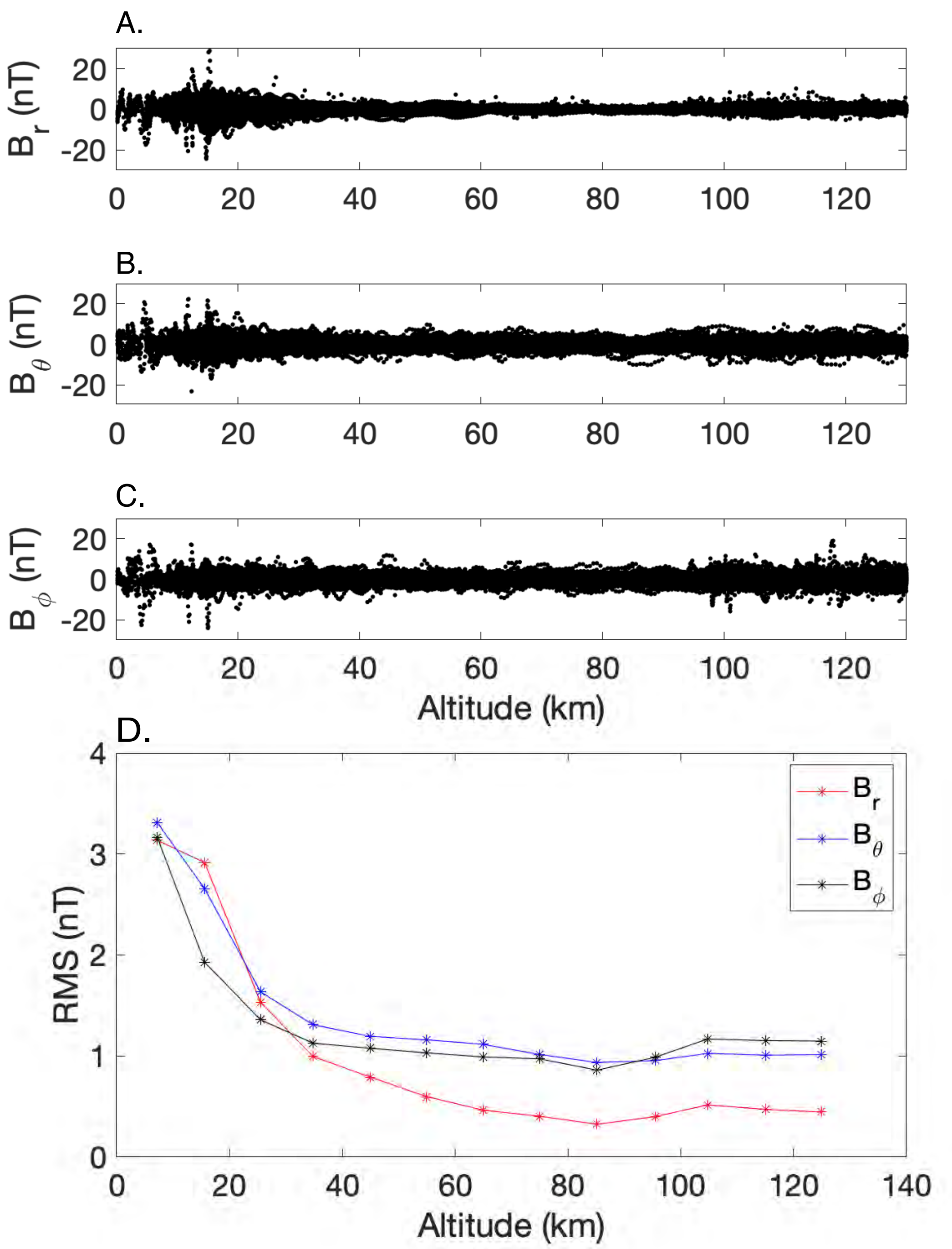

Fig. S2. (A) Radial (B_{r}), (B) colatitudinal (B_{θ}), and (C) longitudinal (B_{ϕ}), magnetic field components of the high-pass-filtered (HPF) data versus altitude. (D) Root-mean-square (RMS) HPF values in 10 km altitude bins. The horizontal components (B_{θ}, B_{ϕ}) show more noise c.f. B_{r}, at altitudes $\geq$ 35 km.

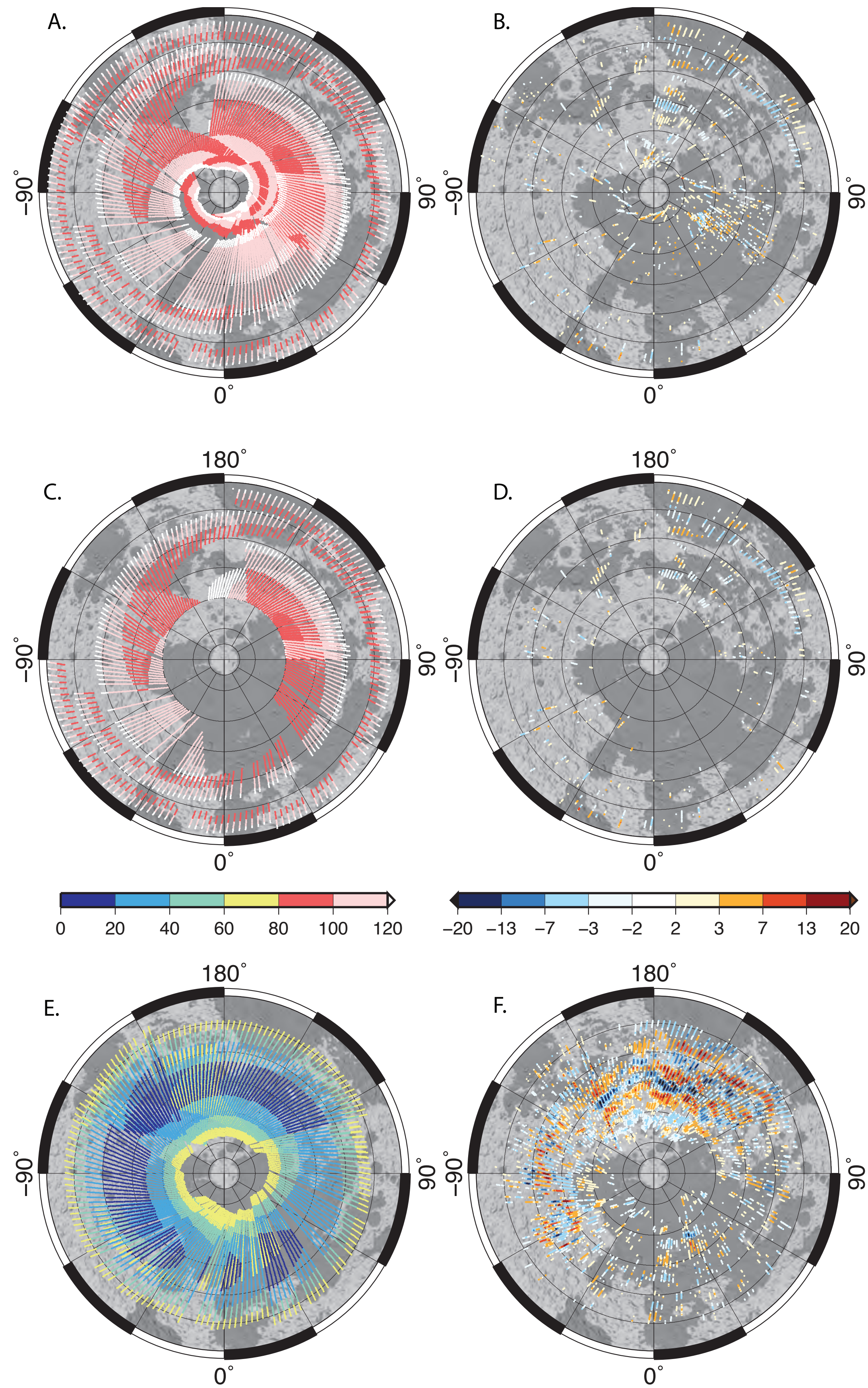

Fig. S3. (A,C,E) Spacecraft altitude (km) and (B,D,F) HPF B_{r} in nT (plotted only where $|B_{\mathrm{r}}| > 2$ nT) for subsets of data for which (A,B) altitudes 80 – 130 km, (C,D) altitudes 80 – 130 km and further restricted to latitudes $<70\,^{\circ}$ N and local times = 6 pm – 6 am, and (E,F) altitude <80 km. Data subsampled by a factor of 3 for plotting. Figure format as in Fig. S1 except that the latitude range is 30 - 90 $^{\circ}$N so that the southernmost limit of the data distribution is seen.

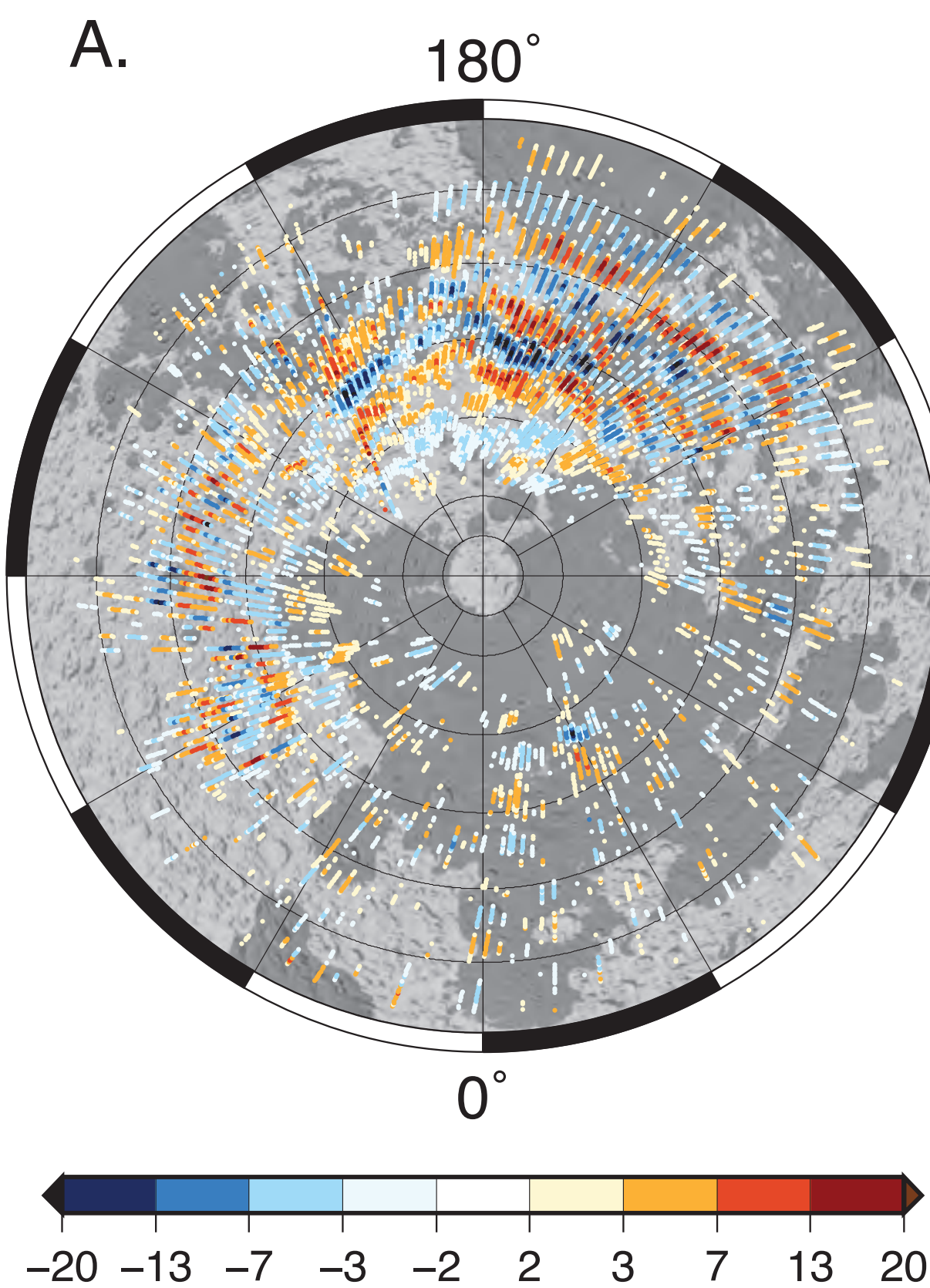

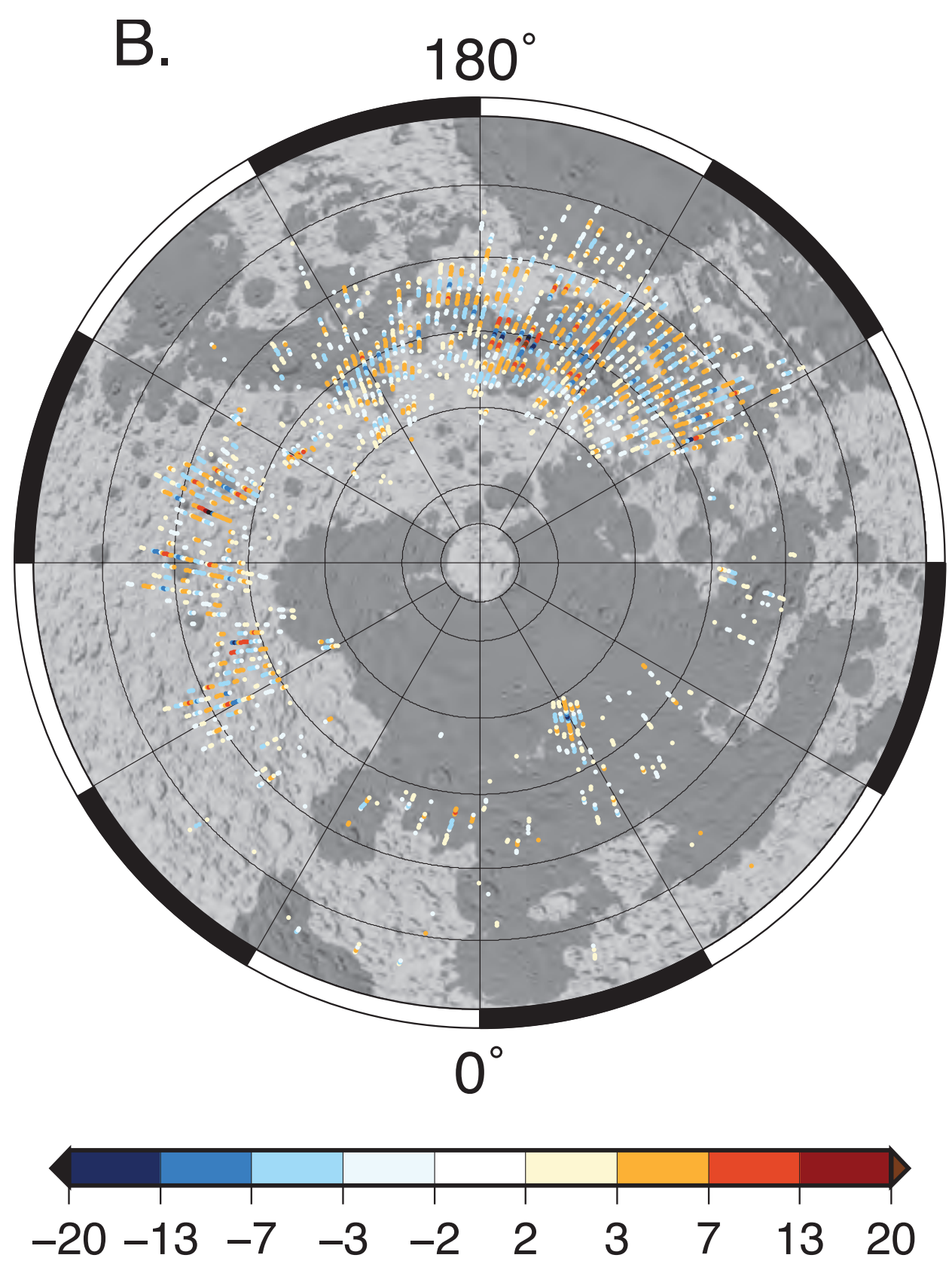

Fig. S4. (A) HPF data used in this study (wavelengths $<\sim$ 800 km retained). (B) Data filtered to retain only wavelengths $<\sim$ 215 km, consistent with the pre-processing steps used in (3, 4). Figure format as in Fig. S3.

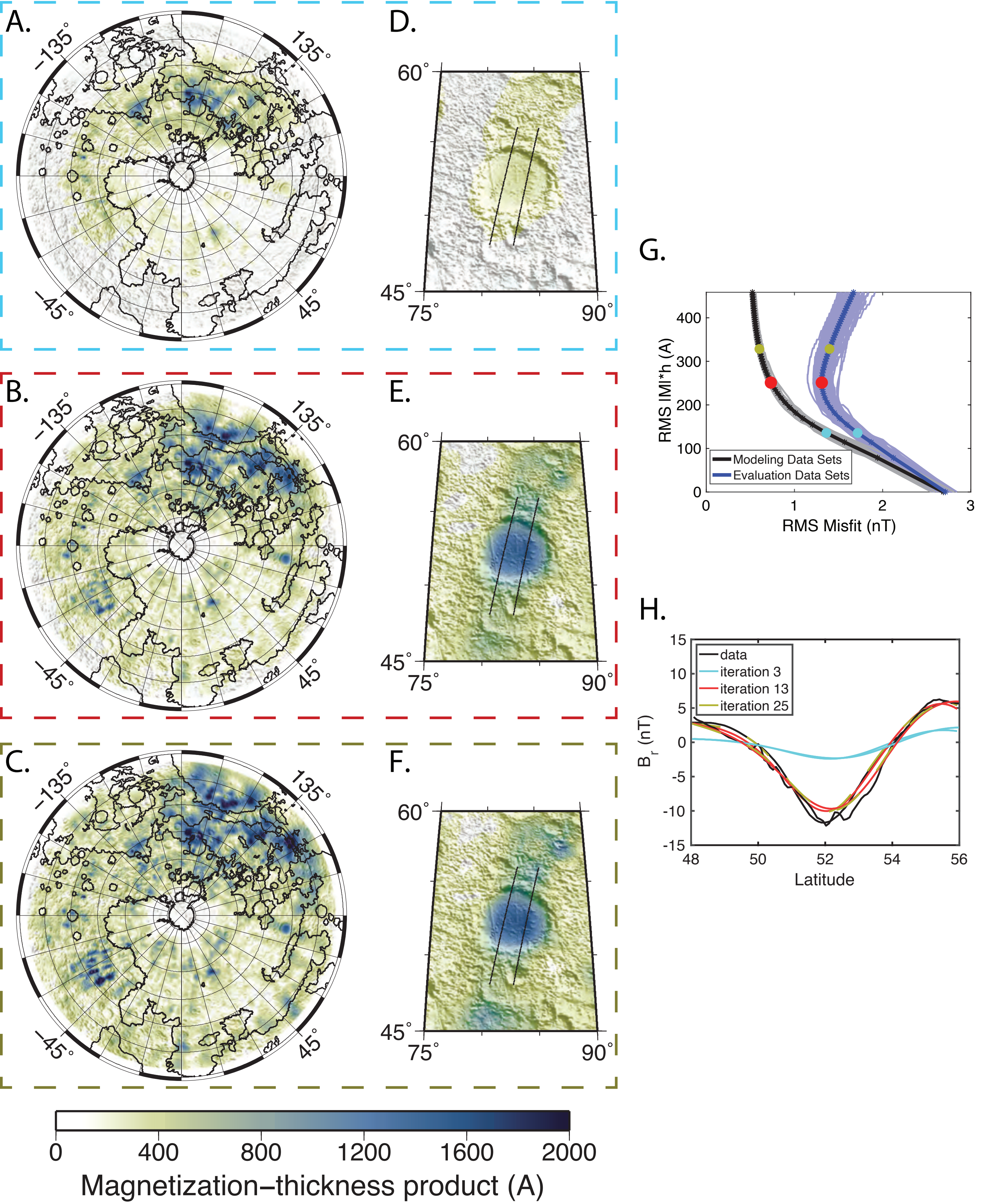
A.
B.
C.
D.
E.
F.
G.
H.
−135°
135°
−45°
45°
60°
45°
75°
90°
RMS |M|*h (A)
RMS Misfit (nT)
Modeling Data Sets
Evaluation Data Sets
B_r (nT)
Latitude
data
iteration 3
iteration 13
iteration 25
0
400
800
1200
1600
2000
Magnetization–thickness product (A)

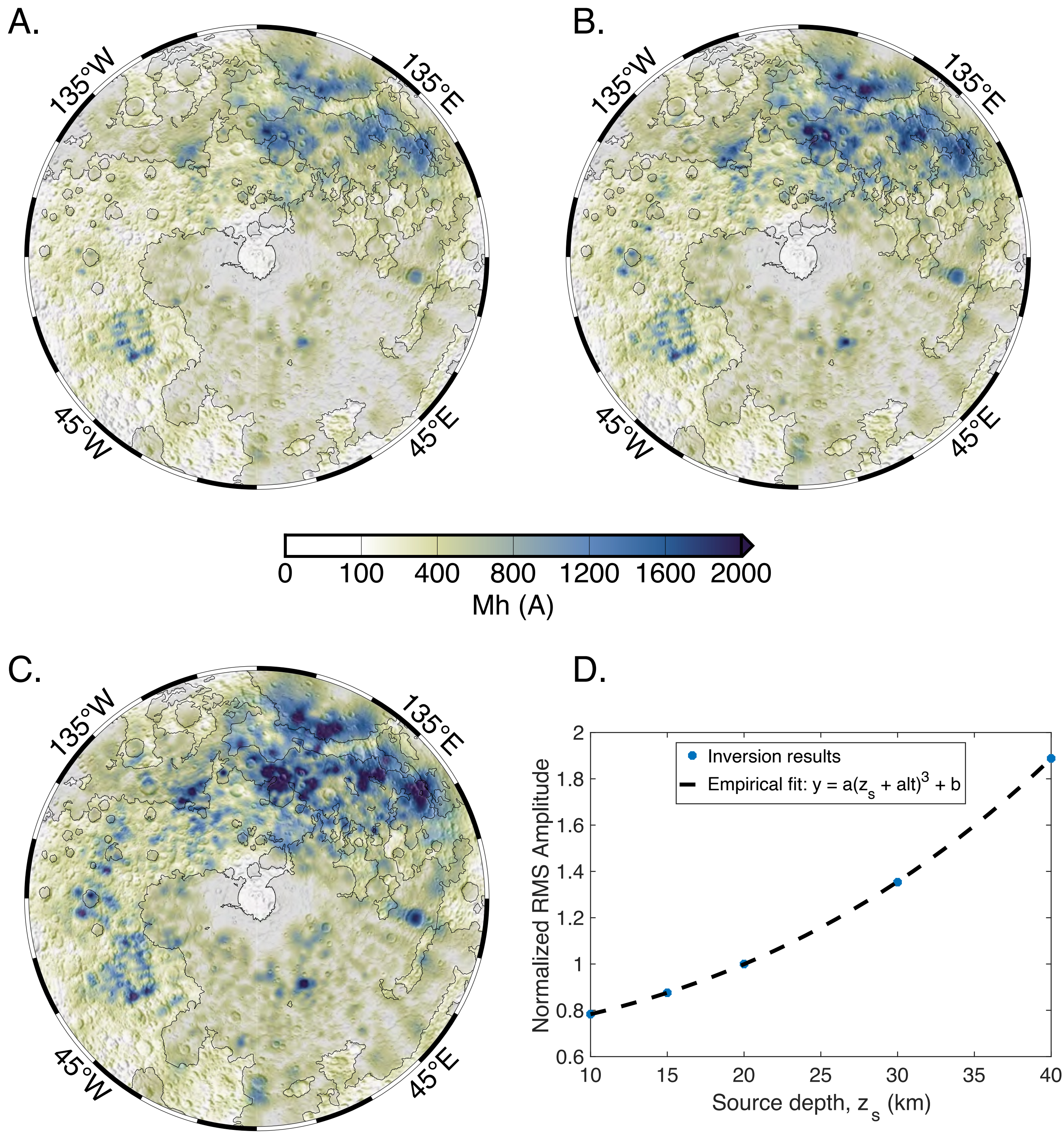

A.
B.
C.
D.
135°W
135°E
45°W
45°E
0
100
400
800
1200
1600
2000
Mh (A)
Inversion results
Empirical fit: y = a(z_s + alt)^3 + b
Normalized RMS Amplitude
Source depth, z_s (km)

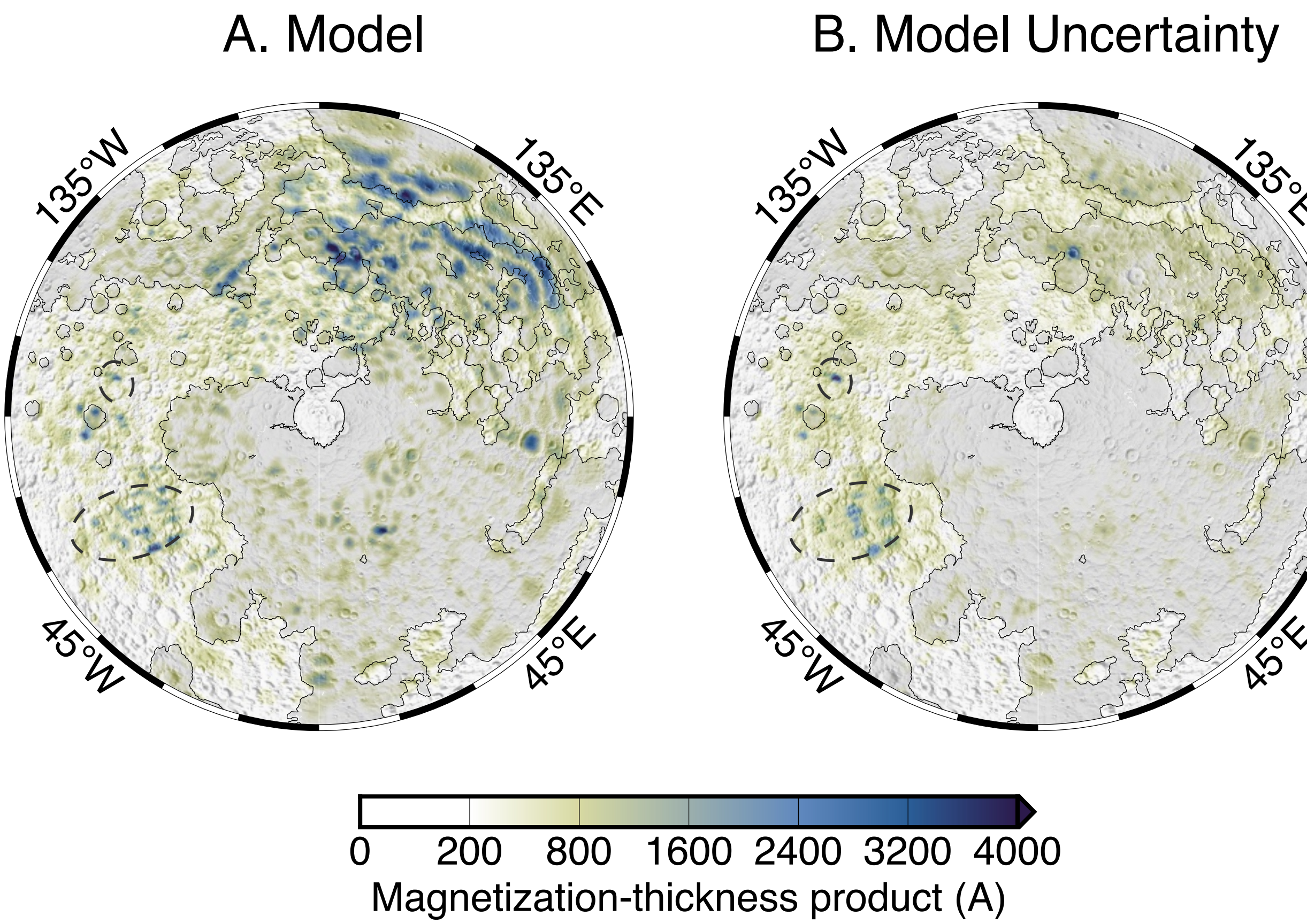
A. Model
B. Model Uncertainty
135°W
135°E
45°W
45°E
135°W
135°E
45°W
45°E
0
200
800
1600
2400
3200
4000
Magnetization-thickness product (A)

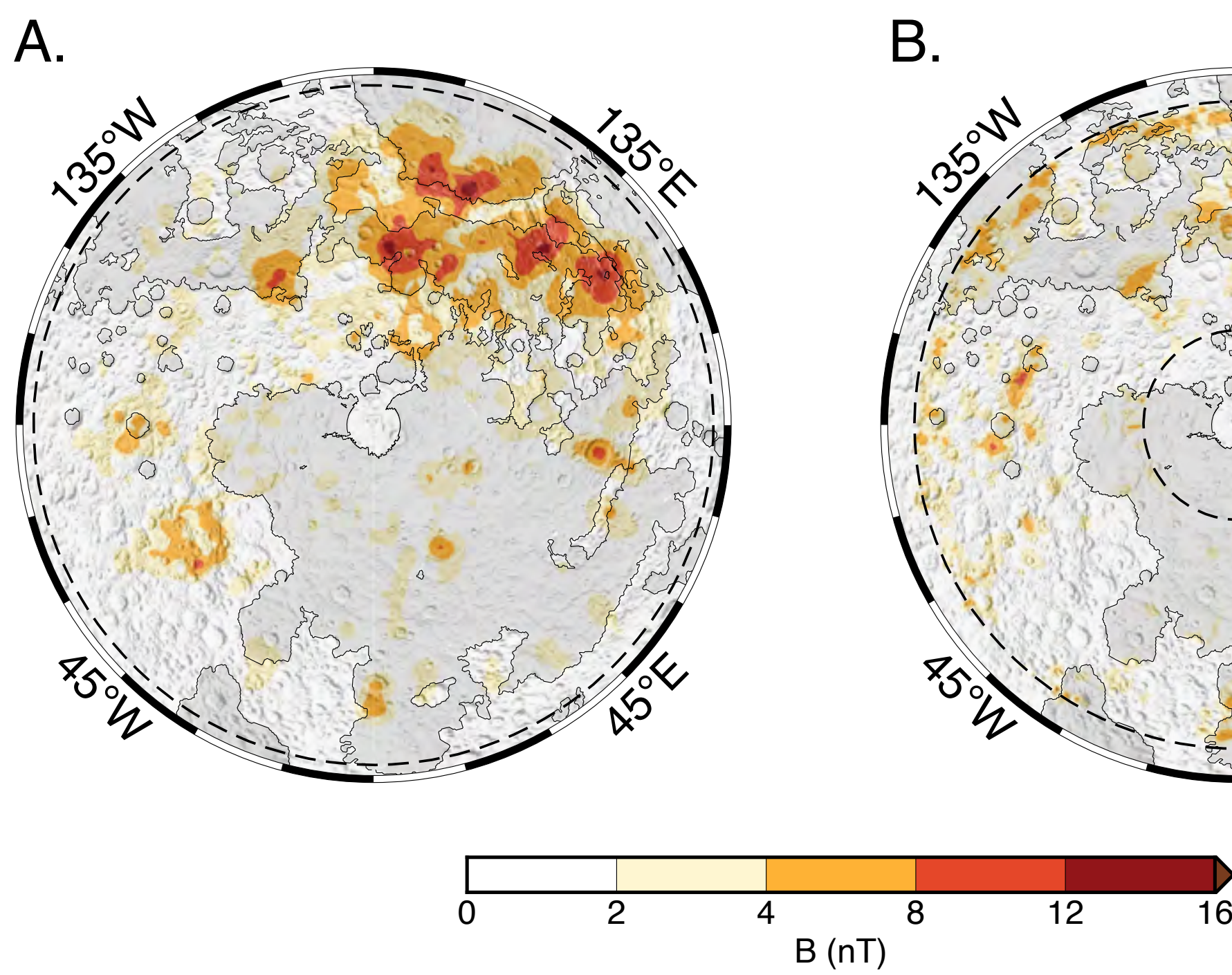

Fig. S8. Comparison of magnetic field amplitude at 40 km altitude for (A) the preferred model in this paper with (B) that of (4). The model in A is resolvable north of the dashed line at 32 °N (Materials and Methods), the modeled region in B is denoted by the dashed lines at 35 °N and 70 °N. The models are compared at 40 km altitude because the inversion approach in (4) means that predicted amplitudes at lower altitudes are dominated by noise. Generally larger model amplitudes as well as some larger spatial scale features are seen in (A) as expected from the differences in the data use in the inversion (Fig. S4). Our model, resolvable north of lacks the edge effects seen at the lower and upper latitudinal extents of the model in (B), e.g. at the lower latitude limit between 0 °E and 45 °E.

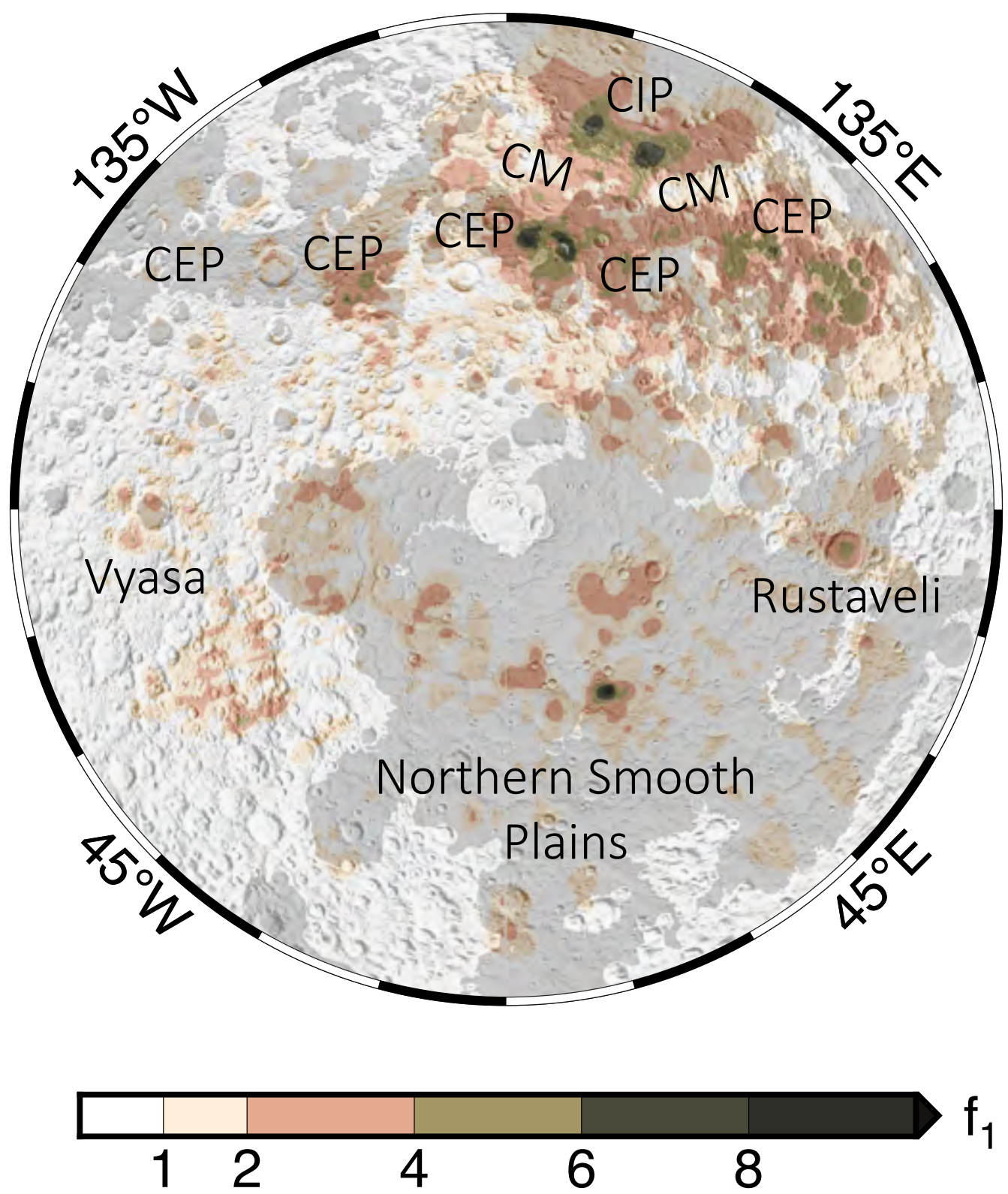

Fig. S9. f_1 as in Fig. 4, but for the crustal thickness model V0 which incorporates lateral variations in density (5).

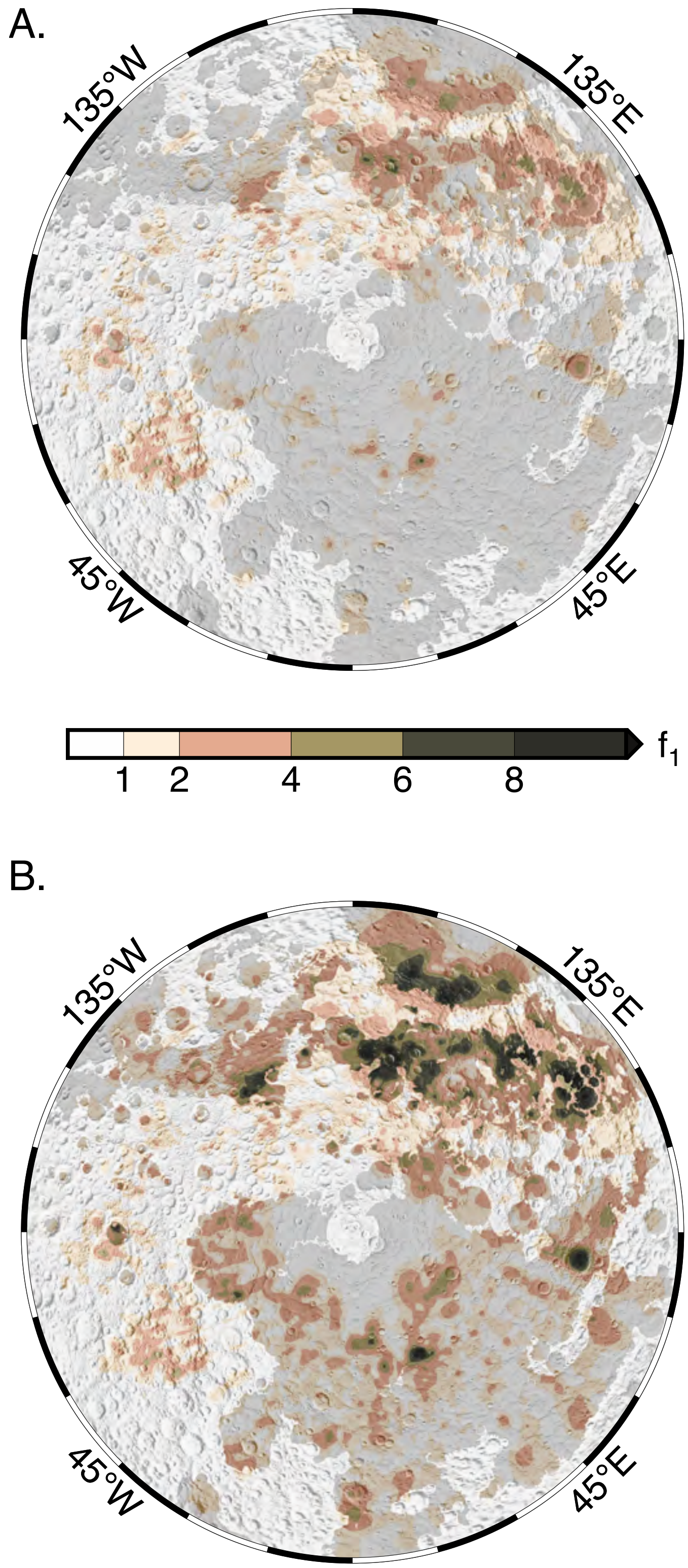

Fig. S10. f_1 for two end-member models for iron content beneath the SP. (A) f_1 computed assuming the wt % Fe beneath the SP is that of the ICP (1.49 wt %), i.e., the SP are a thin veneer everywhere covering a crustal column that is almost entirely ICP units. (B) f_1 assuming that no ICP units underly the SP, i.e. the crustal column contains 0.6 wt % Fe.

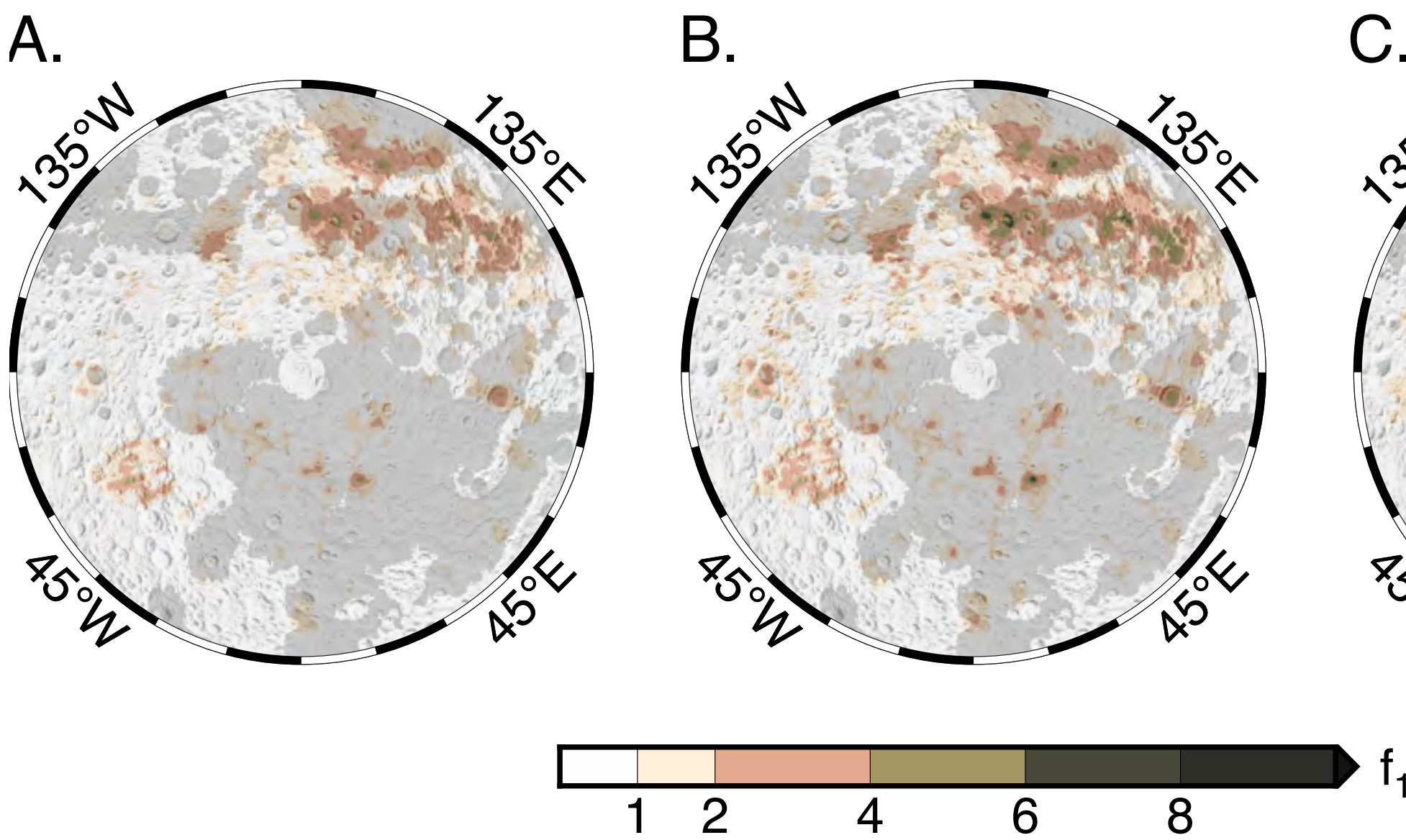

Fig. S11. f_1 for different source depths, Z_S. (A) Z_{S} = 10 km, (B) Z_{S} = 20 km (as in Fig. S4), (C) Z_{S} = 40 km. f_1 scales with Z_{S} by the factor shown in Fig. S6D.

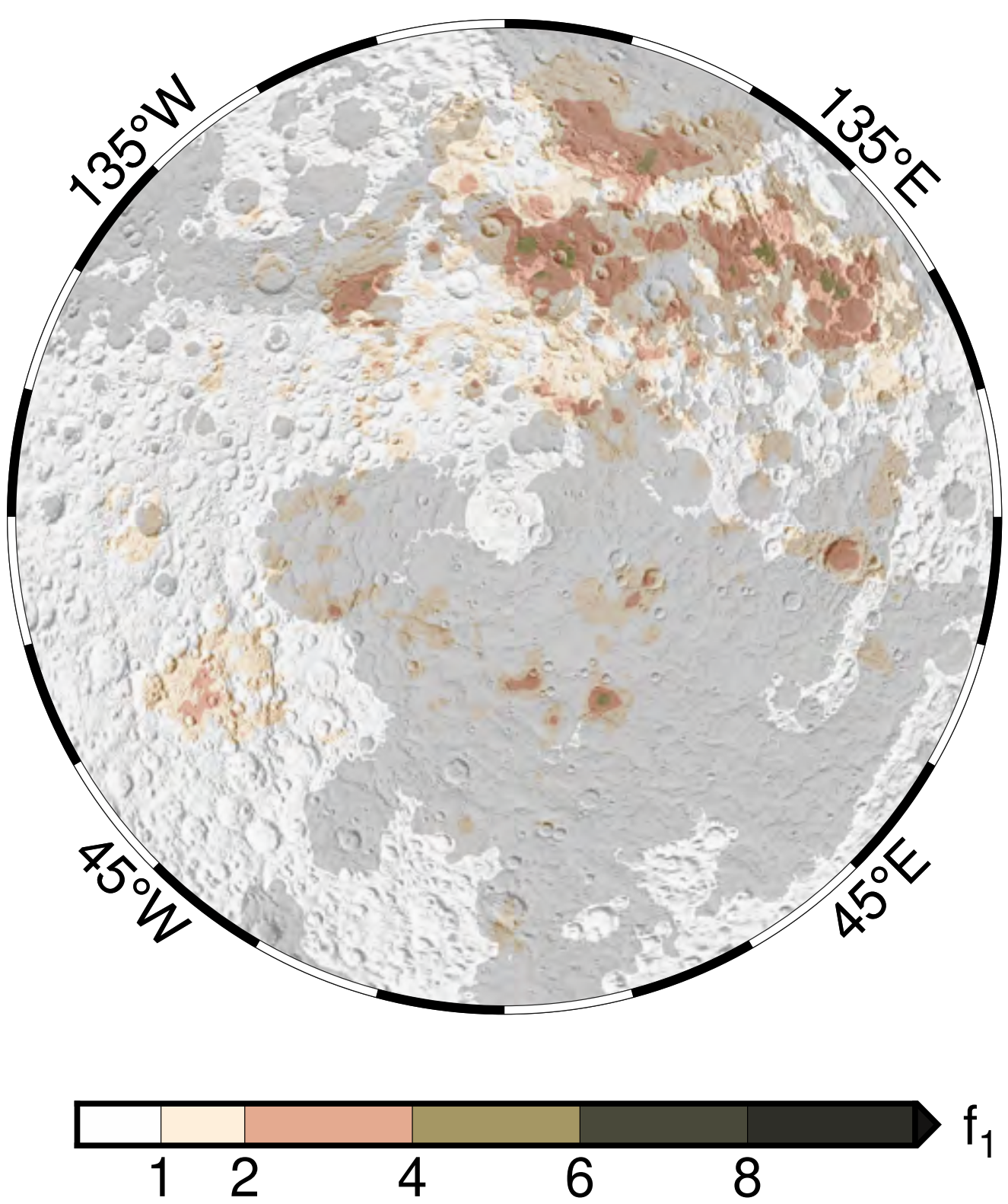

Fig. S12. f_1 for the Mh model that is the 17th percentile, i.e. the average model (Fig. 2A) minus the model uncertainty (Fig. 2B).

Table S1. Comparison of Data and Modeling Approaches.

	H16 [a], H18 [b]	Johnson et al., 2018	This Study
**Data Selection**			
Longitude, ϕ	90–270 °E [a]<br>270–90 °E [b]	All	All
Latitude, λ	35–75 °N	n/a [c]	n/a [c]
Altitude	n/a [d]	$<$ 100 km	$<$ 130 km
Local times	no selection [e]	15:40–08:20	15:40–08:20
Quiet data	hand-editing	no ICME[f]	no ICME, activity $<$ 95<br>additional high alt. criteria [g]
# orbits	95 [a]<br>106 [b]	427	555
**Pre-processing**			
Step 1:	Subtract quadratic	KT15[h]	KT17[i]
Step 2:	Subtract 5° mean	HPF[j] $>$ 0.075Hz	HPF $>$ 0.005 Hz
Wavelengths retained	<215 km	<515 km	<770 km
**Model Parameterization**			
ESD spacing	$\Delta\lambda$= 1°, $\Delta\phi$=2° [k]	20 km [l]	25 km [l]
ESD depth	20 km	20 km	20 km
Direction assumed?	yes	no	no
Bounds, ϕ	overlapping 30 ° sections	0–360 °E	0–360 °E
Bounds, λ	35–80 °N	30–90 °N	30–90 °N
Buffer for S. latitude	no	yes	yes
**Inversion approach**			
model region	8 local inversions	single inversion	single inversion
model uncertainty	no	no	yes, bootstrap

Notes:
[a,b] H16, H18 correspond to Hood, 2016, and Hood et al., 2018 respectively.
[a,b] Selection used in H16 or H18 respectively; the same for both if neither [a] nor [b] specified.
[c] Latitude range of data results from altitude selection criterion, and spans 30–80 °N.
[d] Altitude range of data results from latitude selection criterion, and is generally $<$ 100 km.
[e] No local time selection applied, but most data on orbits used are night-time.
[f] ICME = Interplanetary coronal mass ejection.
[g] See Text S1, Figures S1 and S2.
[h,i] KT15, KT17 correspond to Korth et al., 2015 and Korth et al., 2017 respectively.
[j] HPF = High pass filtered.
[k] Equivalent to 43 km along track and 43 km across track at 60 °N.
[l] Icosahedral grid.

Table S2. Magnetic susceptibility and density data for selected meteorites.

Meteorite	SubClass	Density (kg m^{-3})	χ (10^{-6} m^3 kg^{-1})	$\log_{10}(\chi)$	χ (SI)	Ref
Coolac	I A	7680	396	2.598	3.04	KP83
Toluca	I A	7160	977	2.990	7.00	KP83
Carbo	II D	7540	636	2.803	4.80	KP83
Carlton	III C	7070	1303	3.115	9.21	KP83
Casas Grandes	III A	7880	1416	3.151	11.15	KP83
Grant	III B	7770	3418	3.534	26.55	KP83
Mungindi	III C	7780	1004	3.002	7.81	KP83
Muonionalusta	IV A	7810	1360	3.134	10.62	KP83
Bohumilitz	I A	7510	855	2.932	6.42	T93
Braunau	II A	7095	705	2.848	5.00	T93
Lenarto	III A	7740	1964	3.293	15.20	T93
Henbury	III A	7820	806	2.907	6.31	T93

χ is the low field susceptibility, KP83 refers to (6) and T93 refers to (7). For the four meteorites from (7) (last 4 rows), Tables 1 and 2 of that reference provide two measurements of χ in SI units, one each taken in labs in Finland and Czechoslovakia. We converted those measurements to $\log_{10}(\chi)$ using the densities provided from each lab, and averaged the $\log_{10}(\chi)$ values (as data for χ are typically log normally distributed) to obtain the values in column 5. From this average we derived χ (10^{-6} m^3, column 4), and we used the averaged density for the two samples (column 3) to then calculate the SI value (column 6).

References

1. H Korth, CL Johnson, L Philpott, NA Tsyganenko, BJ Anderson, A dynamic model of Mercury's magnetospheric magnetic field. *Geophys. Res. Lett.* **44**, 10147 – 10154 (2017).
2. CL Johnson, et al., Low-altitude magnetic field measurements by MESSENGER reveal Mercury's ancient crustal field. *Science* **348**, 892–895 (2015).
3. LL Hood, Magnetic anomalies concentrated near and within Mercury's impact basins: Early mapping and interpretation. *J. Geophys. Res.* **121**, 1016–1025 (2016).
4. LL Hood, JS Oliveira, V Galluzzi, DA Rothery, Investigating sources of Mercury's crustal magnetic field: Further mapping of MESSENGER magnetometer data. *J. Geophys. Res.* **123** (2018).
5. M Beuthe, B Charlier, O Namur, A Rivoldini, TV Hoolst, Mercury's crustal thickness correlates with lateral variations in mantle melt production. *Geophys. Res. Lett.* **47**, e2020GL087261 (2020).
6. IT Kukkonen, LJ Pesonen, Classification of meteorites by petrophysical methods. *Bull. Geol. Soc. Finland* **55**, 157—177 (1983).
7. M Terho, LJ Pesonen, IT Kukkonen, M Bukovanská, The petrophysical classification of meteorites. *Stud. Geophys. Geod.* **37**, 65–82 (1993).
